# A comprehensive phylogeography of the *Hyles euphorbiae* complex (Lepidoptera: Sphingidae) indicates a ‘glacial refuge belt’

**DOI:** 10.1038/srep29527

**Published:** 2016-07-21

**Authors:** Michael B. Mende, Manuela Bartel, Anna K. Hundsdoerfer

**Affiliations:** 1 Senckenberg Naturhistorische Sammlungen Dresden, Museum für Tierkunde, Königsbrücker Landstraße 159, D-01109 Dresden, Germany; 2 Georg-August-Universität Göttingen, Johann-Friedrich-Blumenbach-Institut für Zoologie und Anthropologie, Abteilung für Morphologie, Systematik und Evolutionsbiologie, Berliner Str. 28, D-37073 Göttingen, Germany; 3 Biodiversität und Klima Forschungszentrum (BiK-F), Senckenberganlage 25, D-60325 Frankfurt am Main, Germany

## Abstract

We test the morphology based hypothesis that the Western Palaearctic spurge hawkmoths represent two species, the Eurasian *H. euphorbiae* and Afro-Macaronesian *H. tithymali*. It has been suggested that these species merged into several hybrid swarm populations, although a mitochondrial phylogeography revealed substructure with local differentiation. We analysed a three-gene mt-dataset (889 individuals) and 12 microsatellite loci (892 individuals). Microsatellite analyses revealed an overall weak differentiation and corroborated the superordinate division into two clusters. The data indicate that the populations studied belong to only one species according to the biological species concept, refuting the opening hypothesis. A future taxonomic revision appears necessary to reflect the division into two subgroups. Ancestral mitochondrial polymorphisms are retained in *H. euphorbiae*, indicating gene flow within a broad ‘glacial refuge belt’ and ongoing postglacial gene flow. Diverse patterns of extensive mito-nuclear discordance in the Mediterranean and the Middle East presumably evolved by more recent processes. This discordance indicates introgression of *H. tithymali*-related mitochondrial haplogroups, accompanied (to a lesser degree) by nuclear alleles, into Italian and Aegean *H. euphorbiae* populations as recently as the late Holocene. The complex mosaic of divergence and reintegration is assumed to have been influenced by locally differing environmental barriers to gene flow.

The discipline of phylogeography has substantially advanced our understanding of the formation of geographically structured diversity within and among closely related species and thus enabled intriguing insights into the origin of species^1^,^2^. While geographic isolation promotes divergence and speciation^2^, gene flow counteracts this process by merging populations through introgressive hybridisation^3^. Phylogeographic studies have found most species of temperate latitudes to show distinct spatial patterns that are ascribed to vicariance during the severe climatic oscillations of the Pleistocene ice ages^2^,^4^,^5^. Distinct entities evolved in isolated glacial refugia, expanded at the beginning of the current interglacial following a few general patterns and generally formed narrow contact and/or hybrid zones upon secondary contact^2^,^5^. However, the process of divergence and reintegration may also lead to a more complex organisation of diversity that challenges a strictly bifurcating and hierarchically structured classification into discrete entities^6^. Introgression between two taxa can proceed differently depending on locally varying circumstances^2^,^7^,^8^ and hybridisation may even give rise to independent evolutionary entities and/or species^3^,^6^,^9^. Furthermore, the assumption that the patterns of spatially distinct entities have generally remained static since their formation in the early Holocene^2^,^5^ has been challenged recently in favour of a more dynamic evolution^7^, especially for climate-sensitive lepidopterans^8^,^10^,^11^,^12^,^13^.

The spurge hawkmoths (*Hyles euphorbiae* complex: HEC) represent a widespread, polymorphic and taxonomically controversial species complex that features enigmatic biogeographic patterns in an incipient stage of speciation in the Western Palaearctic^14^,^15^,^16^,^17^. We excluded the Sardo-Corso-Balearic endemic *H. dahlii* and non *Euphorbia*-feeding *H. zygophyllii* from this study, although they are formally included in the HEC^18^. They show distinct and fixed morphological characters (see images in Kitching^19^). Geographic distributions of larval and adult colour pattern morphotypes^17^,^20^ (Fig. 1; for illustrations also see Danner *et al*.^21^), as well as initial molecular studies based mainly on mitochondrial genes^14^,^15^,^16^,^18^, led several authors (e.g. Hundsdoerfer *et al*.^14^,^15^,^16^,^18^,^20^, Pittaway^17^) to hypothesise that the Eurasian *H. euphorbiae* and the Arab-Afro-Macaronesian *H. tithymali* hybridise in the Mediterranean. They readily hybridise in captivity without any evidence for reduced hybrid fitness, i.e. postzygotic isolation (see summary in Hundsdoerfer *et al*.^14^). Although a possible prezygotic isolation barrier caused by differing ‘calling’ times has been reported (*H. euphorbiae* females tend to attract males with pheromones and mate before midnight, *H. tithymali* after midnight^22^), larval morphotypes showing intermediate features^17^,^20^ have been found in nearly the entire Circum-Mediterranean^20^. They resemble captive-bred hybrids^23^ and indicate amalgamation of the two species into several hybrid swarm populations^17^,^20^,^23^ (Fig. 1). They have been formally named^19^,^21^ based on morphology: on Crete and the Dodecanese Islands, *H. cretica* (Fig. 1: *CRE); in Italy south of the northern Apennines, *H. euphorbiae* var. ‘*grentzenbergi*’ (*EIT, *CIT, *SIT, *SIC, *PAN, *LAM); on Malta, *H. sammuti* (*MAL); in the coastal and montane areas of the Maghreb, *H. tithymali mauretanica* (*TUN, *MOR); and on the coast of north-western Spain, *H. t. gallaeci* (Galicia: *GAL). A morphological transition zone of putative hybrid origin is also reported for the relict populations on the Arabian Peninsula^17^ (Fig. 1, north of YEM). Based on derived adult and larval morphology, an endemic species, *H. robertsi*, has been described for the Middle East and Central Asia^19^. The populations sampled in this study are currently treated under five species names by Kitching^19^: *H. cretica*, *H. euphorbiae*, *H. robertsi*, *H. sammuti* and *H. tithymali*). However, the two underlying assumptions of this paper are that (i) *H. cretica* and *H. sammuti* are hybrids of *H. euphorbiae* and *H. tithymali*, and (ii) *H. robertsi* is a local form of *H. euphorbiae*. We thus only use the two species names *H. euphorbiae* and *H. tithymali*.

Due to the complexity of mitochondrial haplotype distribution found in a first phylogeographic study on the HEC^16^, the two main haplogroups detected in *H. euphorbiae* and *H. tithymali* are referred to as ‘*euphorbiae*’ and ‘*tithymali*’. Not all individuals of *H. euphorbiae* have ‘*euphorbiae*’ mitochondria, since the ‘*tithymali*’-related haplogroup ‘*enigmatica*’ also occurs in this species^16^. The Maltese population (*MAL) consists of a mixture of individuals bearing three different mitochondrial haplogroups^14^,^15^,^16^,^18^. Apart from both ‘*euphorbiae*’ and ‘*tithymali*’ (thus corroborating a hybrid origin^14^,^18^), it also contains a more distantly related haplogroup, ‘*melitensis*’, that has been assumed to represent the relict of an ancient endemic entity. Distinct, although ‘*tithymali*’-related haplogroups, ‘*italica*’ and ‘*cretica*’, predominate in southern Italy (corresponding to populations *CIT, *SIT, *SIC in this study) and southern Aegean Islands (*CRE) suggesting these areas represent glacial refugia of distinct taxa rather than hybrid zones.

By analysing museum specimens from Italy (corresponding to populations NIT, *EIT, *CIT, *SIT, *SIC in this study) and southern Aegean Islands (*CRE), Mende & Hundsdoerfer^12^,^13^ found that mitochondrial haplogroups attributable to *H. euphorbiae* (‘*euphorbiae*’ and ‘*enigmatica*’) were once mixed among the endemic ‘*tithymali*’-related haplogroups, ‘*italica*’ and ‘*cretica*’. It was only during the Twentieth Century that ‘*italica*’ increased in frequency in Italy from below 60 percent of the total population to near fixation. This was ascribed to genetic drift resulting from habitat destruction and/or to a spread of this haplogroup through the population facilitated by climate warming^12^. This challenges the existence of refugial, long-term isolated and endemic entities and argues for the hypothesis of *H. euphorbiae* and *H. tithymali* hybridisation in these areas^12^,^13^.

The drawbacks of phylogeographic inference from mtDNA alone are known^24^, but in combination with nuclear markers the data can provide valuable understanding of a species’ evolutionary course^25^ and patterns of introgressive hybridisation^26^,^27^. Therefore, we used 339 individual sequences of the ~2300 bp Hundsdoerfer *et al*.^16^ COI/II mitochondrial HEC dataset, and supplemented it with 550 individuals. In addition to this expanded mitochondrial dataset, we also analysed 12 microsatellite loci in 892 individuals to provide a comprehensive overview of the evolutionary history of the Western Palaearctic HEC. We discuss the main causes that could account for the complex phylogeographic patterns observed. The hypothesis (H_1_) to be tested is that the HEC populations marked with a * in Fig. 1 (morphologically intermediate) evolved by hybridisation of two species, *H. euphorbiae* and *H. tithymali*. We formulate the null hypothesis (H_0_) as follows: the HEC consists of a single gene pool without species boundaries within the Western Palaearctic.

## Methods

### Sampling

**Figure 1:**
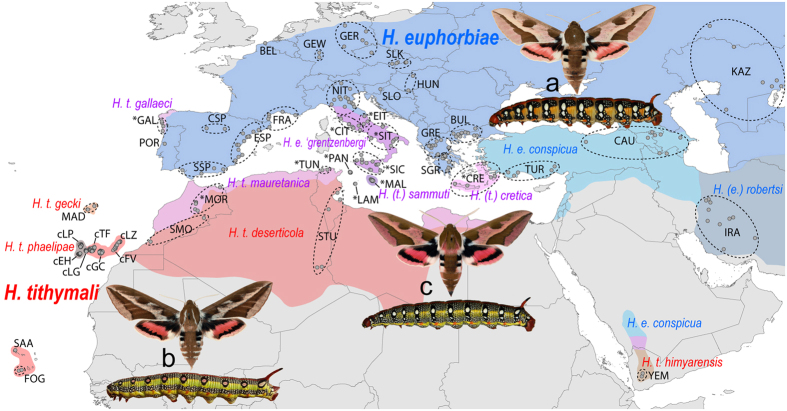
Distribution of the traditionally defined HEC taxa based on a compilation of larval and adult colour pattern morphotypes. (Distribution data combined from Pittaway^17^, Danner *et al*.^21^, Hundsdoerfer *et al*.^20^; map: ArcGIS version 10, ESRI, http://www.esri.de; all inset fotos by MBM, except for larva (b) by AKH). Shades represent *H. euphorbiae* (bluish), *H. tithymali* (reddish) and putative hybrid populations (purple) according to our hypothesis H_1_. Grey dots mark sampling localities; dashed ellipses and abbreviations indicate pooled populations (* = putative hybrid populations). BEL: western Belgium, BUL: Bulgaria to northeastern Greece, CAU: Transcaucasia, cEH: El Hierro, cFV: Fuerteventura, cGC: Gran Canaria, *CIT: western central Italy, cLG: La Gomera, cLP: La Palma, cLZ: Lanzarote, *CRE: southern Aegean Islands (Dodecanese and Crete), CSP: central Spain, cTF: Tenerife, *EIT: eastern central Italy, ESP: eastern Spain, FOG: Fogo, Brava (Cape Verde), FRA: southern France, *GAL: Galicia, GER: eeastern Germany to northern Czech Republic, GEW: western Germany, GRE: northwestern Greece, HUN: Hungary, IRA: Iran, KAZ: Kazakhstan to southern Russia, *LAM: Lampedusa, MAD: Madeira, *MAL: Malta, *MOR: northern Morocco, NIT: northern Italy, *PAN: Pantelleria, POR: Portugal, SGR: southern Greece, *SIC: Sicily, *SIT: southern Italy, SLK: southern Czech Republic to Slovakia, SLO: Slovenia, SMO: southern Morocco, SSA: Santo Antao (Cape Verde), SSP: southern Spain, STU: southern Tunisia, *TUN: northern Tunisia, TUR: southern Turkey, YEM: Yemen. *Insets*: Typical moth and final instar (L5) larva morphotypes: *H. euphorbiae* larva with two large eyespots and a posterior coloured wedge per segment; moth with bright median stripe on forewing which extends to costal margin (a). *H. tithymali* larva with lower eyespots absent and a yellow-coloured lateral band crossing the segments; moth with broad darkened subcostal area of forewing, and veins in postmedial area and borders of tegulae often whitish (b). Putative hybrid population larva with lower eyespots reduced in size and a yellow-speckled lateral band; moth with intermediately darkened costal margin (c).

Larval tissue or legs (if specimens were reared to or caught in the adult stage) of 898 specimens were collected from various localities covering virtually the entire Western Palaearctic distribution range of the HEC (Fig. 1 and Supplementary Table S1) and stored in pure ethanol or dry at −80 °C. For a statistically robust implementation of some analyses, we had to pool samples from several localities to work with a total of 43 populations (Fig. 1); however, we avoided pooling across morphological transition zones or potential geographic barriers, e.g. sea straits.

### Laboratory techniques

In addition to DNA from 339 specimens that had already been isolated for mtDNA sequence analysis^16^, we extracted DNA from further specimens using the NucleoSpin Tissue Kit (Macherey-Nagel) or the InnuPREP DNA Mini Kit (Analytik Jena). Three mitochondrial genes (partial COI/II and interposed tRNA-leu; 2284 bp in total) were amplified using the primers given by Hundsdoerfer *et al*.^14^,^15^ and established protocols^16^. The PCR products were sequenced on an ABI 3130xl (Applied Biosystems) and sequences aligned and proof-read using BioEdit 7.0.9.0^28^.

An unexpected diversity of distinct mitochondrial haplogroups was detected in *H. euphorbiae* (see Hundsdoerfer *et al*.^16^ and this study). Hence, we had to confirm that the sequences correspond to those of transcribed mitochondrial genes rather than nuclear pseudogenes (“numts”) by performing cDNA transcription of mRNA. RNA was extracted from frozen tissue of specimens bearing a haplotype of the ‘*euphorbiae*’ (Supplementary Table S1: #5651, 5699), ‘*melitensis*’ (#5658, 5674), ‘*enigmatica*’ (# 5663, 5666) and ‘*italica*’ (#5649) mtDNA haplogroups with the ‘Perfect Pure RNA Cell & Tissue Kit’ (5Prime) in a dedicated RNA laboratory. RNA concentration was measured with a NanoDrop ND-1000 (Thermo Scientific). The ‘RevertAid First Strand cDNA Synthesis Kit’ (Thermo Scientific) was used to synthesise cDNA from 5 μg RNA according to the manufacturer’s manual; however, with an extended DNA digestion at 37 °C for 1.5 h. A short fragment of COI (fragment ‘B’) and of COII (‘L’) was amplified for haplogroup assignment (for primers and protocol of fragment amplification, see Mende & Hundsdoerfer^12^). All fragments matched the respective sequence obtained from mtDNA amplification.

We analysed the six microsatellite loci described by Mende *et al*.^29^ (Hti62, Hti63, Hti65, Hti66, Heu72, Heu76) and another six loci developed by Hundsdoerfer *et al*.^30^ (Hyti13, Hyti14, Hyti22, Hyti41, Hyti49, Hyti50) using protocols adapted for primers with dye labels (see below). Further described loci were discarded because they did not reliably cross-amplify (Hyti1, Hyti5, Hyti6, Hyti7, Hyti28) and/or presented substantial scoring problems due to a high number of barely distinguishable alleles caused by indels in the flanking regions (Hti67, Heu68). PCR was performed in a volume of 20 μl containing 1 U *Taq* polymerase (Bioron), PCR reaction buffer with a final concentration of 2.5 mM MgCl_2_ (Bioron), 0.2 mM of each dNTP (Fermentas), approximately 10–50 ng DNA template and infrared dye labelled forward and plain reverse primers. In addition, the reactions for loci developed by Hundsdoerfer *et al*.^30^ contained 4 μg bovine serum albumin (BSA) and a raised final concentration of 3.75 mM MgCl_2_. Primers were partly multiplexed and concentrated as follows: 0.18 μM Hti62 + 0.25 μM Hti63 + 0.38 μM Heu76; 0.33 μM Hti65 + 0.13 μM Hti66 + 0.13 μM Heu72; 0.13 μM Hyti13 + 0.2 μM Hyti41 + 0.2 μM Hyti49; 0.01 μM Hyti14; 0.1 μM Hyti22; 0.03 μM Hyti50. The PCR of loci from Mende *et al*.^29^ comprised initial 5 min at 94 °C, 39 cycles of 30 s at 94 °C, 45 s at 56 °C and 1 min at 72 °C and final 20 min at 72 °C. For loci from Hundsdoerfer *et al*.^30^, the protocol was modified to 94 °C for 5 min, 36 cycles of 30 s at 94 °C, 30 s at 56 °C (for loci Hyti13, Hyti41, Hyti49; or 62 °C for Hyti14; 53 °C for Hyti22; 61 °C for Hyti50) and 1 min at 72 °C and final 5 min at 72 °C. The PCR products were analysed on an ABI 3130xl sequencer using an internal size standard (LIZ 600, Applied Biosystems). Fragment size was determined manually with the software PeakScanner 1.0 (Applied Biosystems).

Previous characterisation of the loci showed that highly variable flanking regions cause allele lengths that were inconsistent with the microsatellite repeat modulus^29^,^30^. Further alleles that were not present in the populations tested in these primer notes were verified to avoid artefacts by exemplarily sequencing homozygote specimens, if present. Nevertheless, given that allele length variation is in large part not solely attributable to repeat motif variation, we refrained from analyses that are based on a model of microsatellite evolution.

### MtDNA sequence analyses

Evolutionary relationships among mitochondrial sequences were evaluated by a median-joining network using Network 4.5.1.0 (Fluxus Technology). Since the analysis is prone to errors in the presence of ambiguities, base positions with >10 ambiguities in the total alignment were deleted, leaving 2070 bp. The few remaining ambiguities at non-informative sites were overwritten to the respective base at their particular locus and those at parsimony-informative sites to the most common base of the corresponding haplogroup. Average p-distances between haplogroups were calculated with Mega 4.0.2^31^ and their geographic distribution was illustrated using ArcGIS version 10 (ESRI). DnaSP 5.0^32^ was used to assess haplotype and nucleotide diversity within the entire HEC and the two clusters inferred by the Structure analysis of microsatellite data (see below).

We tested for signals of population size changes for each haplogroup with Arlequin 3.1^33^. Demographic and/or range expansions (or selective sweeps) are expected to generate an excess of recent and thus rare mutations, leading to a unimodal mismatch distribution and negative values of neutrality tests. Instead, a stationary population size (or balancing selection) would result in a multimodal mismatch distribution and a bottleneck in positive neutrality tests. We also inferred past dynamics of effective female population size (*N*_ef_) for each haplogroup by means of a Bayesian coalescent approach with 3 × 10^8^ Markov chain Monte Carlo (MCMC) iterations (of which 1.25 × 10^3^ were sampled for ‘*euphorbiae*’ and ‘*tithymali*’, 2.5 × 10^3^ for ‘*italica*’ and 5 × 10^3^ for the remaining haplogroups) using Beast 1.8.0^34^,^35^. The simulation (data were partitioned into three codon positions) was based on the following assumptions: the HKY nucleotide substitution model, a strict molecular clock with a substitution rate μ = 3.54 × 10^−8^ per year based on a standard COI divergence rate of 3.54% per million years for univoltine insects^36^ (i.e. μ = 1.725 × 10^−8^ per generation; see Schenekar & Weiss^37^) and a generalised average of two HEC generations per year^17^,^21^. We assured that the MCMC chains converged by means of effective sample size values for the parameters, i.e. ESS > 200, and visually. The results were visualised by means of skyline plots using Tracer 1.6^38^.

### Microsatellite marker analyses

We tested for linkage equilibrium (LE) in each population containing ≥15 individuals using Genepop ‘on the web’ 4.2^39^ with default Markov chain (MC) parameters and for Hardy-Weinberg equilibrium (HWE) using Arlequin with 100,000 MC and 10,000 dememorisation steps. Significance of the results was evaluated after Bonferroni correction. Micro-Checker 2.2.3^40^ was used to test for null allele bias. We found no consistent significant deviation from LE between any pair of loci. Excessive deviations from HWE in >50% of the populations were only found for the loci Hyti49, Hti62 and Heu76 (Supplementary Table S2). All deviations could be explained by the presence of null alleles (Supplementary Table S2), which are commonly encountered in lepidopterans^29^.

Pairwise differentiation (F_ST_) between HEC populations was calculated with Arlequin (with 20,000 permutations to test for significance; populations containing <4 individuals, i.e. GEW, SLO, POR and *LAM, excluded). We tested for correlation between genetic and geographic distance by performing a Mantel test implemented in IBDWS 3.23^41^ using the F_ST_ matrix computed by Arlequin, a geographic distance matrix calculated from the average population coordinates (Supplementary Table S1) by means of GDMG 1.2.3^42^, and 30,000 randomisations to test for significance of the correlation. Additionally, we computed a Mantel correlogram with 10,000 permutations and 49.4 ± 0.6 population pairs per distance class using PASSaGE 2^43^.

To provide a robust assessment of population structure in the HEC, we adopted two strategies:

1. A hierarchical AMOVA was performed using Arlequin to test the data support (based on an average over 12 loci) of *a priori* population structure hypotheses. According to the traditional species distribution ranges and H_1_, “*H. euphorbiae*” was defined to contain the populations IRA, KAZ, CAU, TUR, BUL, GRE, SGR, *CRE, HUN, SLK, GER, BEL, FRC, ESP, CSP, SSP, *GAL, NIT, *EIT, *CIT, *SIT, *SIC and *PAN and “*H. tithymali*” STU, *MOR, SMO, cLZ, cFV, cGC, cTF, cLG, cEH, cLP, MAD, SAA, FOG and YEM (Fig. 1). The populations *MAL and *TUN were excluded. It has been postulated for over ten years^14^,^15^ that these populations are of hybrid origin, due to the discrepancy between conserved characters from adult morphology (“*H. euphorbiae*-look” of moths) and mitochondrial assignment (*H. tithymali* or a mixture including *H. tithymali*). The hypothesis for the *a priori* group definitions is thus based on other sources of characters than the ones tested, to avoid circular argument.
2. Analysis of the data without defining *a priori* groups. We performed Bayesian cluster analyses using Structure 2.3.2^44^ and the R package Geneland 4.0.3^45^,^46^. Both searched for clusters that best fit the assumption of HWE and LE. We corrected for the presence of null alleles (see above) by coding them as recessive in Structure^47^ and/or by means of null alleles frequency estimation along the clustering algorithm in Geneland^48^. We assumed the admixture model with correlated allele frequencies in Structure^49^ and uncorrelated allele frequencies in Geneland so as not to overestimate *K*^45^. The spatial clustering algorithm in Geneland was further provided with information of coordinates in decimal degrees (Supplementary Table S1) with an uncertainty value of 10 degrees (this large grid was chosen to avoid bias via coordinates, since the moths fly far). Both programs computed ten replicate runs for each value of *K* = 1–20 with 10^6^ MCMC iterations of which the first 2.5 × 10^5^ were discarded as burn-in (2.5 × 10^3^ of the 10^4^ sampled iterations in Geneland). We reran the Geneland computation with the same parameters except for *K* being fixed for the best two values inferred from the first computation (see below) to obtain more reliable estimates of individual cluster assignment^46^. The optimal number of clusters was inferred using the *ΔK* method of Evanno *et al*.^50^ [rejecting *K* = 1 using mean lnP(D) values] for the Structure analysis and from the posterior probability distribution given by Geneland. Individual probabilities of cluster membership of the replicate run with the highest posterior probability were displayed as barplots with Distruct 1.1^51^ for the optimal partition *K* = 2 and the second most probable partition *K* = 3 (see Results) to assess consistency of cluster boundaries and to reveal potential substructure (see e.g. Fontaine *et al*.^52^). Geographic distribution of the *K* = 2 clusters was also illustrated using ArcGIS 10. In addition, we evaluated the variance of the data using a principal component analysis (PCA) as implemented in the R package Adegenet 1.3-6^53^, a method that is less prone to sample size bias than Bayesian cluster analyses.

Differentiation of Structure and Geneland clusters was inferred using a locus-by-locus AMOVA (as a weighted average over loci) using Arlequin. We assessed genetic diversity within the entire HEC and the Structure clusters by counting the numbers of alleles and computing allelic richness (mean of all loci) with Fstat 2.9.3.2^54^. Private alleles were summarised using Convert 1.3.1^55^ and observed and expected heterozygosity (both summed over all loci) were calculated using Arlequin. For these analyses, all samples of a geographically defined population were collectively assigned to a cluster according to the predominant assignment of its individuals.

MtDNA sequences: GenBank accession number AJ749460-469/472-486/488-494/499-500/503-521/524-528/532/535/537-563/565-567/570-575/577, FN386562-563/600-604, FR839374-532/534-577/579-580/582-583/585-615, European Nucleotide Archive (ENA) accession numbers for new sequences: LT595043-594, available at http://www.ebi.ac.uk/ena/data/view/LT595043-LT595594. MtDNA sequence alignment: Figshare doi: 10.6084/m9.figshare.3438317. Microsatellite genotype table: see Supplementary Table S1.

## Results

### Mitochondrial phylogeography and demographic history

**Figure 2:**
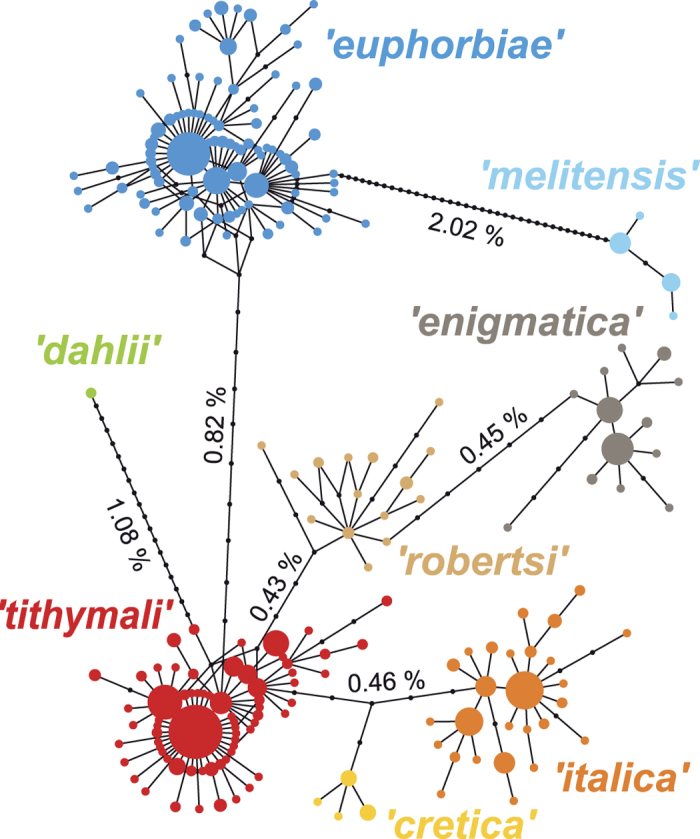
Haplotype network of mitochondrial COI/II sequences. Size of haplotype circles reflects sample size and black nodes represent missing haplotypes. The seven haplogroups are named according to Hundsdoerfer *et al*.^16^ and uncorrected p-distances are given between them.

**Figure 3:**
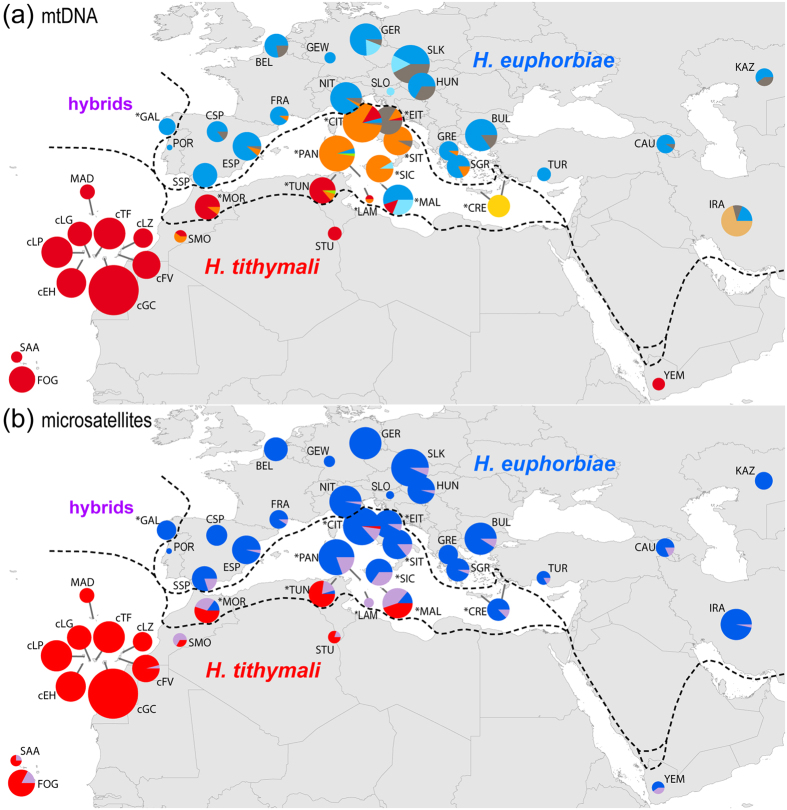
Geographic distribution of mitochondrial haplogroups (a) and microsatellite clusters (b) in a graph illustrating the hypothesis H_1_ formulated by morphology. Dashed lines separate the two traditionally defined species and enclose the areas with larvae of intermediate morphology, representing putative hybrid swarms (see Fig. 1; map: ArcGIS version 10, ESRI, http://www.esri.de). Colours in (**a**) correspond to Fig. 2. Colours in (**b**) correspond to cluster membership according to the optimal (*K* = 2) partition by Structure (see Fig. 7a): *blue* = pure *H. euphorbiae*; *red* = pure *H. tithymali*; *purple* = admixed ancestry (<80% cluster membership; see Randi^81^). Size of pies reflects sample size. Population abbreviations correspond to Fig. 1 (* = putative hybrid populations).

The network analysis of mitochondrial sequences corroborated the previously found division of the HEC into seven haplogroups^16^ (Fig. 2). Additionally, a specimen each from Pantelleria (*PAN) and northern Tunisia (*TUN), both showing the local population’s morphology, were found to carry a haplotype of the distinct Sardo-Corso-Balearic endemic *H. dahlii* (Figs 2 and 3a; the haplotype sequence is identical with *H. dahlii* GB Acc. No. AJ749458).

We detected three haplogroups, ‘*euphorbiae*’, the less frequent ‘*enigmatica*’ and the very distinct ‘*melitensis*’, in the entire Eurasian range of ‘pure’ *H. euphorbiae* (POR, SSP, CSP, ESP, FRA, BEL, GEW, GER, NIT, SLO, SLK, HUN, GRE, SGR, BUL, TUR, CAU, KAZ, IRA), whereas none of the three occurs in the range of the Afro-Macaronesian *H. tithymali* (Fig. 3a; SAA, FOG, cLP, cEH, cLG, cTF, cGC, cFV, cLZ, MAD, SMO, STU, YEM). Notably, ‘*enigmatica*’ is connected to the ‘*tithymali*’ haplogroup via ‘*robertsi*’ in the network (Fig. 2). The ‘*robertsi*’ haplogroup is restricted to Iran, IRA (in the current study; it may extend further east in unsampled populations), where it is mixed with ‘*euphorbiae*’ and ‘*enigmatica*’ (Fig. 3a) in the species *H. robertsi* (according to current taxonomy^19^, but *H. euphorbiae* according to our assumption, see introduction). In contrast, the range of *H. tithymali* (see above) and continental African putative hybrid populations (*MOR, *TUN) is predominantly occupied by a single haplogroup, ‘*tithymali*’. This is absent from the range of morphologically ‘pure’ *H. euphorbiae*; its sole occurrence on the European mainland is in the putative hybrid populations of Central Italy (*CIT, *EIT).

Populations considered being of hybrid origin based on their intermediate larval morphology show remarkably different compositions of mitochondrial haplogroups (Fig. 3a; populations marked with a *). The north-western Spanish population (*GAL) was analysed with molecular methods for the first time in this study and was found to contain only ‘*euphorbiae*’ haplotypes, in contrast to its taxonomic status as *H. t. gallaeci*. Malta (*MAL) was corroborated as consisting of a mixture of haplotypes belonging to ‘*tithymali*’ and two haplogroups, ‘*euphorbiae*’ and ‘*melitensis*’, that also occur in mainland *H. euphorbiae*. In contrast, the other Mediterranean putative hybrid populations are dominated by haplogroups that are equally very closely related to ‘*tithymali*’ and each other (Fig. 2): the Apennine Peninsula (*CIT, *SIT), Sicily (*SIC), and Pantelleria (*PAN) by ‘*italica*’ and the southern Aegean Islands (*CRE) by the endemic ‘*cretica*’ (Fig. 3a). In addition, ‘*italica*’ occurs more frequently and is more widespread in the African populations *MOR and *TUN than was previously found. Additionally, ‘*italica*’ were also found in the supposed non-hybrid *H. tithymali deserticola* of southern Morocco, SMO (Fig. 3a). A few individuals bearing ‘*italica*’ haplotypes were also detected in areas of Mediterranean Europe assigned to morphologically ‘pure’ *H. euphorbiae* (ESP, FRA, GRE, SGR). In turn, the *H. euphorbiae* haplogroups ‘*euphorbiae*’, ‘*enigmatica*’ and ‘*melitensis*’, show occasional occurrences among the dominant ‘*italica*’ in Southern Italy (*CIT/*PAN, *SIT or *SIC respectively).

**Figure 4:**
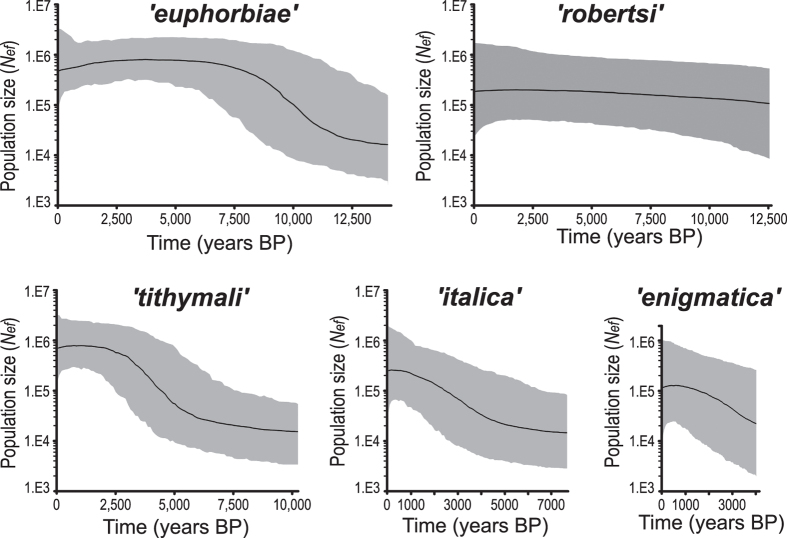
Bayesian skyline plots (BSPs) showing past population size dynamics for mitochondrial haplogroups. The black line indicates the median estimate of effective female population size *N*_ef_ through time, the grey area reflects the 95% highest posterior density (HPD) limits. BSPs for ‘*melitensis*’ and ‘*cretica*’ are uninformative due to a very short time to coalescence (>1,000 years BP) and are thus not shown.

Unimodal mismatch distributions of mitochondrial haplotypes and significantly negative neutrality test results unequivocally support a scenario of demographic and/or spatial expansion for the ‘*euphorbiae*’, ‘*italica*’, ‘*robertsi*’, and ‘*tithymali*’ haplogroups, while support for this scenario is not clear for ‘*cretica*’, ‘*enigmatica*’ and ‘*melitensis*’ (Table 1 and Supplementary Fig. S1). According to the Bayesian skyline plot, the median estimate of population size of the ‘*euphorbiae*’ haplogroup increased substantially at the onset of the Holocene from approximately 11,000 to 8,000 years before present (BP). The increase becomes significant, i.e. when the 95% highest posterior density (HPD) limits loose overlap with older estimates, at about 6,500 years BP (Fig. 4). The following comparably constant plateau appears as a slight decrease in the last ~3,000 years (Fig. 4), but is insignificant and may be an artefact due to pooled sampling from a structured population^56^. The median estimate of the ‘*tithymali*’ and ‘*italica*’ haplogroups started to increase considerably later at about 5,000 years BP (Fig. 4). The expansion becomes significant for ‘*tithymali*’ at about 3,000 years BP. It nearly becomes significant for ‘*italica*’ only after 1,000 years BP, but the 95% HPD limit overlap remains at every time, thus ‘*italica*’ only shows a trend of expansion. The skyline plot of ‘*enigmatica*’ at best reveals low population growth since coalescence at around 4,000 years BP, but due to the high overlap of the 95% HPD limits the population size could also have remained stable. In contrast, the population size of ‘*robertsi*’ is estimated to have remained nearly constant throughout the Holocene (Fig. 4).

### Microsatellite marker analysis (in comparison with mtDNA)

**Figure 5:**
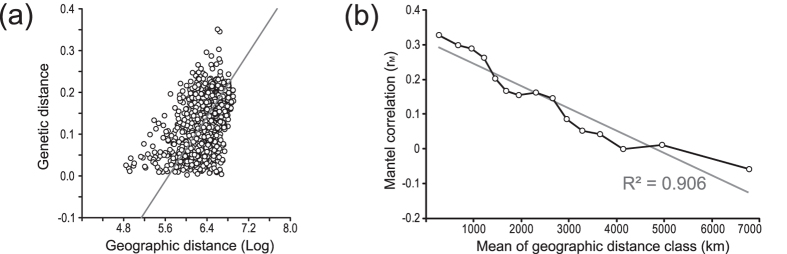
Relationship between geographic and genetic (microsatellites) distance in the entire HEC. Mantel test (**a**): Pairwise comparisons of genetic (F_ST_; see Supplementary Table S3) and geographic distance (log-transformed to account for the two-dimensional distribution of sample sites) between the populations of the HEC. Mantel correlogram (**b**): Mantel correlation of pairwise genetic distance from populations of the entire HEC plotted in geographic distances classes with linear trend line (in grey) and its coefficient of determination given.

Nuclear genetic and geographic distances between populations are significantly correlated across the HEC’s entire range (r_m_ = 0.4276; p < 0.0000; Fig. 5a). Accordingly, 18.3 percent (i.e. r_m_^2^) of the genetic divergence is explained by geographic distance. The Mantel correlogram reveals a continuous and approximately linear decrease of Mantel correlation, i.e. a decrease in genetic similarity, with increasing geographic distance (Fig. 5b).

The result of the hierarchical AMOVA (average over 12 loci) is that 9.9% (*p* < 0.00001) of the total variability in the dataset is found between the two groups of populations defined *a priori*, supporting the division into *H. euphorbiae* and *H. tithymali* (sensu H_1_). Between populations within these groups we found 6.2% (*p* < 0.00001) of the total variability, i.e. less than between the two *a priori* defined groups. However, the greatest part of the variability in the dataset (83.8%) was found between individuals (all values; *p* < 0.00001).

**Figure 6:**
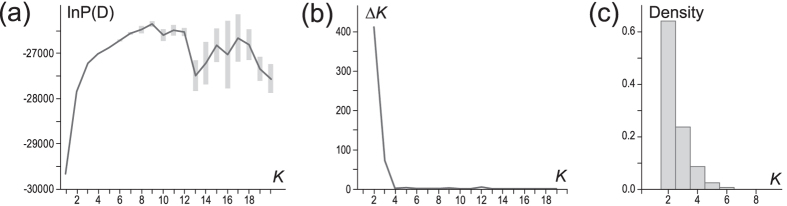
Estimated probabilities for the number of microsatellite clusters. Structure analysis: Mean (±SD) log posterior probabilities (**a**) and ∆*K* values (**b**) for ten replicate runs of each number of clusters *K* = 1–20. Geneland: Posterior probability distribution of number of clusters (*K*) shown for the optimal out of ten replicate runs (**c**).

**Figure 7:**
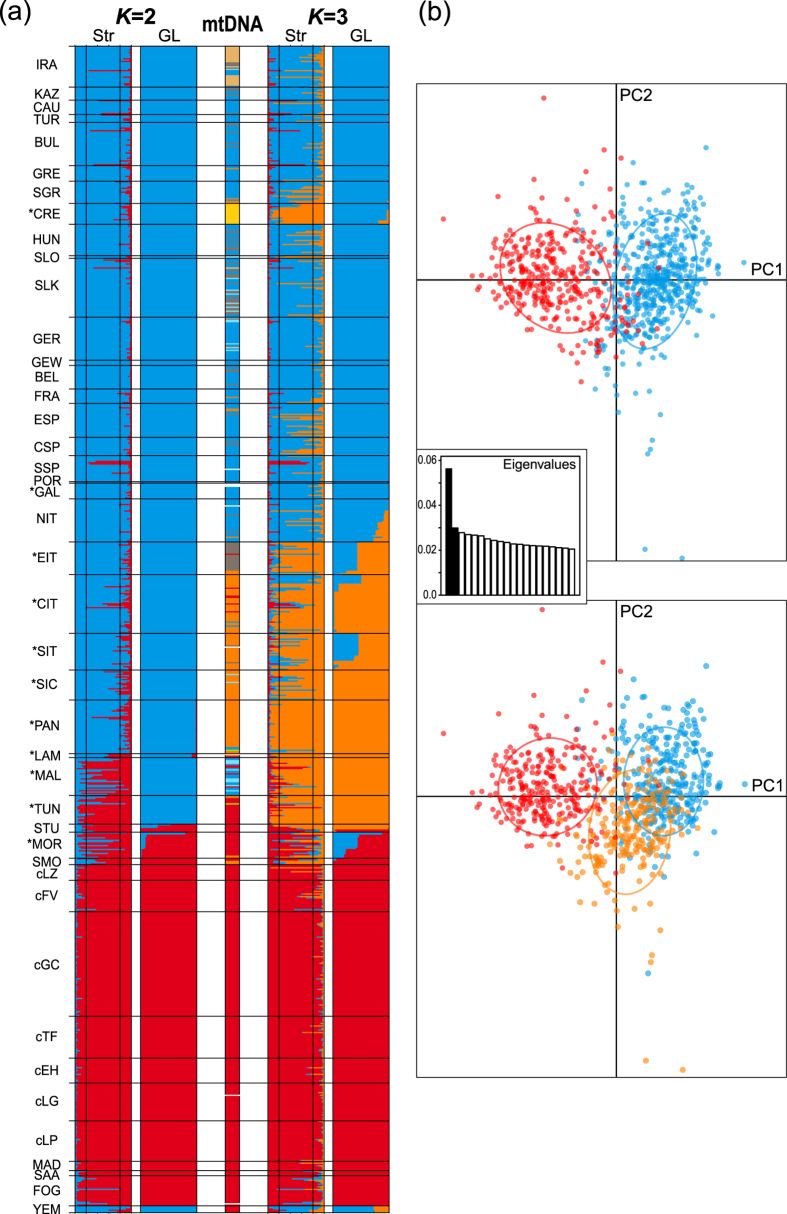
Estimated population structure based on microsatellite data according to Bayesian cluster analyses (with mitochondrial haplogroup assignments for comparison) (**a**) and according to a principle component analysis (PCA) (**b**). Bayesian cluster analyses (**a**): Individual cluster assignment probabilities from the Structure (Str) and Geneland (GL) analyses for *K* = 2 (corresponding to *H. euphorbiae* and *H. tithymali*) and *K* = 3. Order of individuals corresponds to that in the Supplementary Table S1; level of 80% of cluster membership is indicated as the threshold for pure and/or admixed ancestry in the Structure bar plots^81^. Population abbreviations correspond to Fig. 1 (* = putative hybrid populations) and colours for mitochondrial haplogroup assignments to Fig. 2 (white = no mtDNA sequence available). PCA (**b**): Dots represent individuals, their colouration and 95% inertia ellipses visualise assignment to the *K* = 2 or *K* = 3. Structure clusters (with all samples of a population collectively assigned to a cluster according to the predominant assignment of its samples). *Inset*: Eigenvalues of the first 20 components with the proportion of total variance scaled on the y-axis.

The Bayesian cluster analyses of the nuclear microsatellite data concordantly reveal a most probable partition of the HEC into two clusters (Fig. 6), which are moderately differentiated (Structure partition: F_ST_ = 0.091; Geneland: F_ST_ = 0.102) and largely congruent between both analyses except for the populations *TUN and *MAL (Fig. 7a; see below). This differentiation is also (albeit not distinctly) reflected in the first component of the PCA (Fig. 7b) and in an increase in pairwise F_ST_ values between populations assigned to the two different clusters (Supplementary Table S3). Remarkably, this differentiation is mainly attributable to only three loci with F_ST_ values above the average (Hti50, Hti63 and Hti65), whereas it is substantially lower for the majority of loci and some values are even insignificant (Supplementary Table S2).

The geographic extents of the two clusters are largely congruent with the traditionally defined ‘pure’ ranges of the HEC’s two main taxa, which is why we refer to them as *H. euphorbiae* (blue) and *H. tithymali* (red) (Fig. 3b). However, at least a few individuals with admixed ancestry are recorded by Structure throughout most parts of both the species’ ranges. Nearly all individuals bearing ‘*tithymali*’ haplogroup mitochondria are assigned to the *H. tithymali* microsatellite cluster, whereas individuals bearing all other haplogroups are assigned to the *H. euphorbiae* cluster (Figs 3 and 7a). Accordingly, the two microsatellite clusters both contain a high number of individuals with private haplotypes (Table 2). Yemeni *H. t. himyarensis* (YEM) represent an unexpected exception to the congruence between microsatellite clusters and ‘pure’ taxa ranges, since they are predominantly assigned to the *H. euphorbiae* microsatellite cluster by both cluster analyses, in conflict with their morphological and mitochondrial affiliations (Figs 3 and 7a). Likewise, the assignment of individuals of the Iranian *H. robertsi* (IRA) (current taxonomy^19^, assumed to be part of *H. euphorbiae* in this study; see introduction) to the *H. euphorbiae* cluster contrasts with their partly ‘*tithymali*’-related ‘*robertsi*’ haplogroup mitochondria. Remarkably, the diversity of both nuclear and mitochondrial markers is distinctly higher in the *H. euphorbiae* than in the *H. tithymali* cluster (Table 2).

The geographic position of the main divide between the two microsatellite clusters falls within the Mediterranean putative hybrid populations, but is inconsistent between the two cluster analyses (Fig. 7a). According to Structure, the African mainland (*TUN, STU *MOR, SMO) and Maltese (*MAL) populations are substantially admixed but predominantly assigned to *H. tithymali*. In contrast, Geneland assigns *TUN and *MAL to *H. euphorbiae*. The putative hybrid populations of Italy (*EIT, *CIT, *SIT, *SIC, *PAN), and the southern Aegean Islands (*CRE) also include an increased number of admixed individuals according to Structure (Fig. 3b). However, they are predominantly assigned to the *H. euphorbiae* cluster by Geneland. Mitochondrial ‘*tithymali*’-related haplogroups ‘*italica*’ and ‘*cretica*’ (Figs 2, 3 and 7a) prevail in these populations. They thus show a considerable mito-nuclear discordance. The *H. euphorbiae* population of southern Spain (SSP) also shows a considerable number of admixed individuals according to the Structure result. In contrast, all individuals from the north-western Spanish *H. t. gallaeci* population (*GAL) are assigned to the *H. euphorbiae* cluster in both analyses, in congruence with mtDNA.

The results of the second most probable partition, *K* = 3, also deserve attention, since they reveal interesting substructure (Figs 5 and 6). The third cluster includes only the majority of individuals from the Mediterranean populations of putative hybrid origin (*CRE, *EIT, *CIT, *SIT, *SIC, *PAN, *LAM, *MAL, *TUN, *MOR). In the *K* = 2 result above, *TUN, *MAL have been assigned inconsistently to *H. tithymali* by Structure, and to *H. euphorbiae* by Geneland. *EIT, *CIT, *SIT, *SIC, *PAN, *CRE fall into *H. euphorbiae* by both analyses, whereby the latter is assigned to the third cluster only by Structure. A weak differentiation of the third cluster is also indicated in the second component of the PCA (Fig. 7b). Except for Malta (*MAL), the geographic extent of the third cluster in Europe according to Structure is congruent with the distribution areas of ‘*tithymali*’ and the related mitochondrial haplogroups ‘*italica*’ and ‘*cretica*’ (Fig. 7a).

## Discussion

### Phylogeographic structure: two hybridising species *H. euphorbiae* and *H. tithymali*?

The analyses of the nuclear microsatellite data concordantly support a superordinate division of the Western Palaearctic HEC into two clusters. These roughly correspond to the two traditionally defined species, the Eurasian *H. euphorbiae* and Afro-Macaronesian *H. tithymali*.

Remarkably, the mitochondrial haplogroups ‘*enigmatica*’, ‘*robertsi*’, ‘*italica*’ and ‘*cretica*’ are almost exclusively restricted to individuals in the microsatellite cluster representing *H. euphorbiae* (and the distribution range enclosed by these samples), whereas they are more closely connected to the main haplogroup of *H. tithymali* in the network of mitochondrial sequences (‘*tithymali*’). These discordances demonstrate an incomplete separation of *H. euphorbiae* and *H. tithymali*. The underlying causes are probably different for each of the haplogroups. The patchy admixture of ‘*tithymali*’-related ‘*enigmatica*’ with ‘*euphorbiae*’ indicates incomplete mitochondrial lineage sorting with a retained ancestral polymorphism in *H. euphorbiae*. This retained mitochondrial diversity in *H. euphorbiae* is accompanied by a higher nuclear diversity compared to the southern *H. tithymali* (Table 2), which thus contrasts with the general expectation of higher diversity in southern latitudes due to continuous residence during the glacial cycles^15^,^57^. In contrast to ‘*enigmatica*’, the (nearly) exclusive prevalence of ‘*italica*’ (with a visible signal of recent growth) and ‘*cretica*’ in their respective geographically confined areas (*CIT, *SIT, *SIC, *PAN and/or *CRE) and their close relation to the ‘*tithymali*’ haplogroup suggest more recent introgressions of these mitochondrial haplogroups into *H. euphorbiae* (see Toews & Brelsford^27^ and Discussion below).

Our data further indicate ongoing nuclear gene flow far beyond the Mediterranean populations of putative hybrid origin. Individuals assessed as admixed by Structure occur throughout nearly the entire Western Palaearctic HEC range (Fig. 3b) and the two clusters are hardly differentiated at the majority of microsatellite loci (Supplementary Table S2). Congruently, the Western Palaearctic HEC taxa lack diagnostic (i.e. fixed) morphological characters^20^ and no clear or biologically meaningful phylogeographic structure has been found by means of ISSR markers^15^ or nuclear genes^18^. Cumulative evidence points to the HEC constituting only a single gene pool, though structured into two main clusters, refuting H_1_ (two species) and confirming H_0_. The populations studied should thus be classified as belonging to a single species according to the biological species concept. Although a division into two clusters corresponding to *H. euphorbiae* and *H. tithymali* is well supported by the microsatellite data, *H. euphorbiae* is not a maternal lineage monophylum (mtDNA), in the sense that the taxon does not contain all descendants of its most recent common ancestor (MRCA).

Accepting the Western Palaearctic HEC as one biological species is not inconceivable, given that HEC moths are considered strong fliers^15^,^21^ and no postzygotic reproductive barriers have been found in hybridisation experiments between different HEC taxa (see Hundsdoerfer *et al*.^14^). Since the microsatellite clusters representing the HEC’s two main Western Palaearctic entities are fairly well allocated between the African and Eurasian continents (Fig. 3b), the major force that probably caused and maintains this differentiation is the geographical divide posed by the Mediterranean Sea. However, this barrier is not impermeable, as indicated by the high amount of admixture in the nuclear genome on both sides at the narrowest sea straits and particularly by the dominance of ‘*tithymali*’-related haplogroups in parts of Southern Europe.

Moreover, we found evidence for mitochondrial introgression from *H. dahlii*, the spurge hawkmoth species endemic to Sardinia, Corsica and the Balearic Islands, which challenges the alleged effective reproductive isolation of this morphologically and genetically more distinct taxon^14^,^17^,^18^,^21^. *Hyles dahlii* individuals have been reported sporadically from the surrounding mainland coasts and interpreted as rare dispersal events^14^,^17^. However, the detection of individuals carrying a *H. dahlii* haplotype in the established HEC populations *PAN and *TUN suggests that dispersal across the Mediterranean Sea and subsequent hybridisation may be more common than presumed. Detecting genetic traces of such hybridisation should be rather unlikely, given the small chance that rare migrants’ genes can establish themselves in an established population due to the principle of ‘high density blocking’^58^. These events probably contribute to the morphological variability in the HEC.

### Evolutionary history of the two subgroups

The phylogeography of European biota, including mobile lepidopteran species, generally reveals well preserved spatial genetic patterns resulting from differentiation in isolated glacial refugia on the Mediterranean peninsulas and subsequent postglacial re-colonisation^2^,^4^,^5^. The HEC data obtained do not corroborate any of these paradigms of distinct entities expanding at the beginning of the current interglacial and forming narrow contact and/or hybrid zones upon secondary contact in Europe. In contrast, *H. euphorbiae* has three (‘*euphorbiae*’, ‘*enigmatica*’, ‘*melitensis*’) mitochondrial haplogroups (Fig. 3a) occurring throughout its range from the Iberian Peninsula to Central Asia, except for Italy and the Aegean Islands, which are occupied by the two geographically confined haplogroups ‘*italica*’ and ‘*cretica*’ (see also Discussion below). Massive interglacial gene flow could explain why the known postglacial re-colonization paradigms^4^,^5^ are not observed (see Petit^59^) and mitochondrial haplogroup diversity is rather maintained throughout the range of ‘pure’ *H. euphorbiae*. The data denote a scenario by which the organisms retreated into a ‘glacial refuge belt’ in the Circum-Mediterranean area with local differentiation and simultaneous ongoing gene exchange. In this scenario, moths would fly between refugia of favourable climatic conditions, maintaining gene flow and thus the geographic distribution of the mitochondrial diversity within this region. The recolonisation of Central Europe would have occurred by the northward expansion of moths carrying this mitochondrial mixture.

Assessments of past population size dynamics clearly indicate an expansion of the ‘*euphorbiae*’ haplogroup, roughly dated to the early Holocene (Fig. 4). Thus, this signal likely predominantly reflects the lineage’s re-colonisation of more northern European latitudes enabled by postglacial warming^4^,^60^. In contrast, the Bayesian skyline plot of ‘*enigmatica*’ indicates at best much lower population growth, in line with the fact that it is mostly at a lower frequency than the dominant haplogroup in the populations in which it occurs. The ‘*enigmatica*’ haplogroup is currently most frequent in some Italian (*EIT) and Central European (SLK) populations, but has no core area. Our augmented dataset further reveals that ‘*melitensis*’, highly divergent from ‘*euphorbiae*’, is not endemic to Malta as previously assumed^16^ but also occurs on Sicily (*SIC) and the European mainland (GER, SLK, SLO). Thus, conclusions about the places of origin of these haplogroups cannot be drawn unequivocally. Due to the principle of ‘high density blocking’^58^, ‘*euphorbiae*’, ‘*enigmatica*’ and ‘*melitensis*’ must all have occurred in founder individuals coming from the ‘glacial refuge belt’ (see above), and simultaneously occupied the new habitats in Central Europe. The larval food plants of the genus *Euphorbia* need open habitats, which would have increased with the spread of Neolithic culture from 6,500 years B.P. onwards^61^. As postulated before^15^, cultivated steppe would have replaced woodland allowing both foodplants and moths to thrive. Interestingly, the timing corresponds exactly to the significant increase of the population expansion signal of ‘*euphorbiae*’ (about 6,500 years BP; Fig. 4).

The currently observed genetic admixture between *H. tithymali* and *H. euphorbiae* and the high dispersal potential of the HEC leads us to postulate a broad ‘admixture belt’ that oscillated north and south with climate changes^16^. Besides Southern Europe, the Maghreb also often served as a source for the postglacial re-colonisation of Europe by numerous other species^57^ (also see Discussion below). The high proportions of ancestral assignment to the *H. euphorbiae* microsatellite cluster of many African samples (Fig. 3b) and the prevalence of intermediate morphotypes in the northern Maghreb^17^,^20^ (Fig. 1) suggest that at least parts of the nuclear identity of the *H. euphorbiae* entity also introgressed into the African HEC populations. Only the Canary and Cape Verde Islands and possibly unsampled (there are recent records of *H. tithymali* from southern Algeria, the Tibesti Mountains in northern Chad^62^ and Mauritania) and/or now extinct southern peripheral populations of the once ‘green’ Sahara^60^,^63^ are and/or may be left as ‘purest’ populations of the *H. tithymali* entity. Thus, the nuclear data denote an area far smaller than that suggested by the exclusive occupation of the entire African range to the Arabian Peninsula by the ‘*tithymali*’ (and closely related ‘*italica*’) mitochondrial haplogroup^16^.

### Patterns of admixture of the two subgroups

#### Southern Europe

Extensive mito-nuclear discordance as observed in the Italian HEC populations (*CIT, *SIT, *SIC, *PAN) is a common result of introgressive hybridisation^27^. Bayesian estimations of demographic changes indicate an expansion of the ‘*italica*’ haplogroup considerably later than the expansion of ‘*euphorbiae*’ (Fig. 4). Furthermore, it has to be noted that the more southern HEC populations may produce more than the assumed two generations per year, which would yield an even more recent time for the ‘*italica*’ expansion. Consequently, this signal probably reflects a replacement of previously present *H. euphorbiae* haplogroups (‘*euphorbiae*’, ‘*enigmatica*’, ‘*melitensis*) in Italy by ‘*italica*’. This is corroborated by an analysis of museum specimens, which showed that this replacement was still in progress during the last century^12^. Furthermore, it is not yet complete, as revealed by the sporadic findings of *H. euphorbiae* haplogroups throughout Italy. Accordingly, ‘*euphorbiae*’ on Malta is not as disjunct from the mainland as previously thought^16^. However, we could not resolve the initial timing of the introgression of ‘*italica*’ into the European *H. euphorbiae* or the haplogroup’s geographic origin. Its present-day main occurrence would suggest that it initially separated from the ‘*tithymali*’ haplogroup only after it became isolated in Italy. However, the more frequent and widespread occurrence of ‘*italica*’ in the Maghreb (dominant in SMO, but also in *MOR and *TUN) than previously found^16^, rather indicates an African origin and a more recent arrival and spread in Italy. Given that ‘*italica*’ was at a frequency below 60 percent of the total Italian population only a hundred years ago^12^, it can likely be assumed to have been at a much lower frequency still, or even absent from Italy at the beginning of its estimated expansion a few to one thousand years ago (Fig. 4).

Interestingly, the evolutionary history of the Aegean Islands population (*CRE) is suggested to parallel the Italian one. A similar mito-nuclear discordance with a mitochondrial haplogroup closely related to ‘*tithymali*’ is observed. Unfortunately, the small sample size of the ‘*cretica*’ haplogroup, which exclusively dominates the Aegean Islands today, does not allow for a conclusive assessment of its past demographic evolution. However, an analysis of museum specimens revealed that ‘*cretica*’ had once also been admixed with ‘*euphorbiae*’ as far south as Crete^13^. Thus, it cannot be ruled out that ‘*cretica*’ may not be endemic to the Aegean Islands but possibly also have originated and still be present in Africa, like ‘*italica*’. However, more samples, e.g. from the relict HEC population in western Egypt^17^ (including museum material to go back in time), are needed to investigate this idea.

Mitochondrial introgression from *H. tithymali*–related haplogroups into the Italian (*PAN, *SIC, *SIT, *CIT) and Aegean Islands (*CRE) populations was evidently accompanied by a parallel nuclear introgression. This is indicated by the geographically congruent prevalence of intermediate morphotypes^20^ (Fig. 1) and a considerable number of specimens that feature a substantial proportion of ancestral assignment to the *H. tithymali* microsatellite cluster according to Structure (Figs 3b and 7a). These populations’ predominant assignment (*PAN, *SIC, *SIT, *CIT, *CRE, together with individuals from *EIT, *MAL and *TUN) to the separate third Structure cluster at *K* = 3 may also be attributable to this introgression (Fig. 7a). The assignment of the Aegean Islands population (*CRE) to *H. euphorbiae* by Geneland conflicts with the assignment to the third cluster by Structure. This is likely explained by Geneland taking geographic coordinates into the calculation into account and thus weighting the result to rather group individuals from closer geographic vicinities. Crete lies nearer to Greece (*H. euphorbiae*) than to the abovementioned populations in the third cluster.

Cluster algorithms are prone to divide a continuum of gradual differences into distinct clusters^64^,^65^. Thus, the third partition (and the incongruent assignment of *MAL and *TUN at *K* = 2) could reflect an artefact caused by allele frequency gradients between the genetically more distinct *H. tithymali* and *H. euphorbiae* populations in Macaronesia and Eurasia, respectively. A linear decrease of the Mantel correlation with geographic distance (Fig. 5b) also suggests that spatially directed clines–which could arise from a dynamic introgression as discussed above^7^–primarily account for the Mantel correlation^66^. However, the interpretation of Mantel tests and correlograms is highly controversial^64^. It cannot be ruled out that the individuals in the third cluster may in fact have an incipient separate identity, since a very weak differentiation is also suggested by the PCA (Fig. 7b). Animal hybrid swarm differentiation has been shown to occur more frequently than previously presumed^3^,^6^, including examples that evolved similarly from the contact between northern and southern taxa in Italy (sparrows^67^, salamanders^9^). Nevertheless, it yet remains a matter of speculation for the HEC, since the already low genetic separation of the two parental entities with our marker set inevitably cannot provide confirmation of a possible hybrid swarm differentiation.

#### Middle East

In the Middle East, suitable HEC habitats were probably well spread across the ‘green’ Sahara and Arabian Peninsula until deserts expanded a few thousand years ago^60^,^63^ and isolated African, Yemeni and Eurasian HEC populations from each other^17^,^20^. Mito-nuclear discordance with assignment to the *H. euphorbiae* microsatellite cluster and the ‘*tithymali*’ mitochondrial haplogroup is observed in Yemen and accordingly with the ‘*tithymali*’-related ‘*robertsi*’ in Iran. However, the lack of an expansion signal for the Iranian ‘*robertsi*’ during the entire Holocene (Fig. 4) implies that this pattern did not evolve by a similarly recent dynamic introgression as for ‘*italica*’. As a further difference, Iranian samples are not morphologically intermediate: morphotypes show high similarities with typical *H. euphorbiae*^17^,^20^ in agreement with the samples’ microsatellite cluster assignment. In contrast, the morphology of Yemeni specimens strongly resembles *H. tithymali*^17^,^20^, whereas their predominant nuclear genetic assignment is to *H. euphorbiae*. The adjacent Saudi Arabian population has morphologically been assigned to *H. euphorbiae*^17^ (Fig. 1) but also features ‘*tithymali*’ mitochondria (Supplementary Table S1) further indicates complex admixture patterns in this region. However, a considerably larger sample size is needed for a more robust assessment of the population genetic structure and its history in this region.

#### Northwestern Iberia

Finally, the Galician population (*GAL) had also been considered a hybrid population based on morphology. Especially the frequent occurrence of horizontally connected upper eyespots in the larvae, a character frequently found on the western Canary Islands^20^, suggests that the population could have been established by occasional *H. tithymali* vagrants or even to represent a relict refuge of *H. tithymali*^68^. However, we found no traces of *H. tithymali* in either molecular marker. This may be due to the low sample size and the already low differentiation of the two microsatellite clusters. Alternatively, colour pattern similarities may not always reliably reflect genomic evolutionary relatedness of populations^26^,^69^ and thus potentially misguide taxonomic inference^16^. The specific larval colour pattern may be caused by a coincidental combination of alleles of genes involved in complex, multi-locus pattern coding^16^ (also see Harbich & Hundsdoerfer^70^) that became frequent in Galicia and on the western Canary Islands, independently of one another.

### Environmental factors potentially affecting phylogeographic patterns

In accordance with the majority of empirical studies, computer simulations reveal that introgression of selectively neutral markers (and especially uniparentally inherited organelle DNA) almost exclusively occurs from the local to the invading taxon due to demographic disparity^71^. However, the opposite is indicated by the ‘*italica*’ skyline plot (Fig. 4) and an increase in this apparently invading haplogroup, shown to have occurred during the last century^12^. This could happen under specific conditions such as disassortative mating, unequal hybrid fitness, or sex-biased dispersal^27^, each in favour of ‘*italica*’ bearing females. However, these hypotheses lack empirical evidence and appear implausible. A possible infection with *Wolbachia*^16^ that favoured the spread of a mitochondrial lineage^72^ should be analysed in more detail. Alternatively, however, there is growing evidence that mitochondrial variants can be adapted to environmental variables such as temperature gradients^27^,^73^,^74^ and computer simulations show that this can result in phylogeographic structure^75^. Accordingly, the historic increase of ‘*italica*’ and fluctuations of its northernmost occurrences correlate well with warming summer temperatures during the last century^12^. Likewise, the estimated expansions of ‘*italica*’ and ‘*tithymali*’ since the late Holocene (Fig. 4) coincided with a linear increase of temperatures in the Mediterranean during the last 8,000 years^76^, suggesting a selective advantage at higher temperatures.

In contrast to dispersal of ‘*tithymali*’ and related mitochondrial haplogroups across the wide Strait of Sicily and Libyan Sea into Southern Europe, this was not observed across the comparatively narrow Strait of Gibraltar, although southern Spain has a potentially suitable climate for these haplogroups^16^. However, we did record nuclear influx from *H. tithymali* in SSP (Figs 3b and 7a). The Gibraltar Strait forms a strong barrier to gene flow for some bat species but not others, not correlating with the species’ dispersal capabilities^77^. Nevertheless, hawkmoths can be carried by winds >5 m/s^78^ and mitochondrial introgressions into Southern Europe could have happened quite recently, so we hypothesise that current wind regimes could have helped shape the genetic structure. Prevailing Levante and/or Poniente winds, which blow east/west (Supplementary Fig. S2a), would impede gene flow across the Gibraltar Strait effectively, whereas the Sirocco appears to promote dispersal across the Central Mediterranean Sea (Supplementary Fig. S2b), as reported for the HEC^79^. Unlike neighbouring populations (*TUN, *PAN, *LAM, *SIC), ‘*italica*’ does not occur on Malta (*MAL). The considerably different mitogenetic composition can be ascribed to the island’s geographic isolation^10^, especially in respect to prevailing winds (Supplementary Fig. S2b). However, wind regimes apparently do not influence gene flow in the same way throughout the range of the HEC. Strong north-eastern trade winds blow almost permanently across the direct route between the African mainland and the Canary Islands. They also blow from north-east to south-west between the individual islands of this archipelago, but their HEC populations show very low overall genetic differentiation.

## Conclusions

A locally varying interplay of geographic barriers and climate variables, as well as incomplete lineage sorting and introgression, have shaped a truly complex phylogeography of this dispersive hawkmoths species complex. Our study highlights the importance of comprehensive sampling and analyses of multiple markers for a conclusive evaluation of a species’ evolutionary history. The data imply that the populations studied constitute a single gene pool, and thus belong to one biological species. Nevertheless, a division into two subgroups is predominant. Our data put forward a new pattern of a ‘glacial refuge belt’ with both local differentiation and ongoing gene exchange. A subsequent oscillating ‘admixture belt’ is postulated to follow climate fluctuations. The dominance of a distinct mitochondrial haplogroup in the Italian HEC would generally be considered to have evolved from long-term processes of vicariance during glacial periods and to have been static since the early Holocene^2^,^5^. However, our data suggest that it could have evolved more recently. The HEC potentially adds to the few examples of mtDNA variant adaptation to climate gradients known so far that challenge the general assumption of neutrality for this most commonly used marker in phylogeography^1^ and molecular taxonomy^80^.

## Additional Information

**How to cite this article**: Mende, M. B. *et al*. A comprehensive phylogeography of the *Hyles euphorbiae* complex (Lepidoptera: Sphingidae) indicates a ‘glacial refuge belt’. *Sci. Rep.* **6**, 29527; doi: 10.1038/srep29527 (2016).

## Supplementary Material

Supplementary Information

Supplementary Dataset Table S1

## Figures and Tables

**Table 1:** Results of mismatch analyses and neutrality tests for the mitochondrial haplogroups in alphabetical order.

	*‘cretica’*	*‘enigmatica’*	*‘euphorbiae’*	*‘italica’*	*‘melitensis’*	*‘robertsi’*	*‘tithymali’*
Model parameters							
Sample size (n)	16	76	281	144	26	22	322
No. of segregating sites (S)	3	17	81	29	4	20	69
Expansion parameter (τ)	0.980(0.340–1.986)	0.850(0.578–1.234)	2.287(1.668–4.047)	2.367(1.059–3.592)	2.406(0.000–4.494)	3.137(1.918–4.175)	2.203(1.156–3.297)
Pop. size before expansion (θ_0_)	0.000(0.000–0.014)	0.000(0.000–0.030)	0.605(0.000–0.956)	0.004(0.000–0.680)	0.002(0.000–0.033)	0.005(0.000–0.735)	0.079(0.000–0.698)
Pop. size after expansion (θ_1_)	99999.000(5.614–99999)	99999.000(10.562–99999)	50.977(12.327–99999)	11.694(4.543–99999)	2.005(1.210–99999)	99999.000(14.234–99999)	16.162(5.261–99999)
Goodness-of-fit test							
Sum of square deviations (SSD)	0.02840	0.01779	0.00017	0.00775	0.10526	0.00501	0.00170
P (sim. SSD ≥ obs. SSD)	**0.10780**	0.03220	**0.89400**	**0.21130**	**0.12630**	**0.43710**	**0.52670**
Tajima’s D	−0.41395	−1.90772	−2.34719	−1.81130	0.30840	−1.63458	−2.37725
P (sim. D < obs. D)	0.37150	**0.00705**	**0.00000**	**0.00820**	0.66660	**0.03430**	**0.00005**
Fu’s F_S_	−0.82161	−6.97073	−26.20079	−18.55541	0.65185	−9.16373	−26.98077
P (sim. F_S_ ≤ obs. F_S_)	0.16590	**0.00145**	**0.00000**	**0.00000**	0.66375	**0.00010**	**0.00000**

Confidence intervals (95%) for model parameters are given in brackets. Values that support a scenario of demographic and/or spatial expansion (insignificant SSD between observed and expected mismatch; significantly negative values for Tajima’s D and Fu’s F_S_) are marked in bold.

**Table 2:** Summary statistics of genetic variation in the entire HEC and microsatellite clusters.

Group	Microsatellites									Mitochondrial sequences					
	*N*_*nu*_	N_A_	N_Ā_	N_pA_	N_pĀ_	AR	H_O_	H_E_	L_HWD_	*N*_*mt*_	N_H_	N_pH_	N_p%_	H_d_	π
entire HEC	892	261	0.293	–	–	15.43	0.383	0.604	11	889	218	–	–	0.965	0.0065
*H. euphorbiae* cluster	549	248	0.452	149	0.271	16.47	0.374	0.571	8	547	152	147	0.967	0.962	0.0071
*H. tithymali* cluster	343	114	0.332	13	0.038	8.41	0.390	0.587	8 (7)	342	71	66	0.930	0.868	0.0028

Samples of populations were collectively assigned to one of the *K* = 2 Structure clusters (see Fig. 7a) according to the predominant assignment of the populations samples; *N*_*nu*_: number of individuals with microsatellite data; *N*_*A*_: total number of alleles (sum of all loci); *N*_*Ā*_: average number of alleles per individual; *N*_*pA*_ and *N*_*pĀ*_: total and average number of private alleles; *AR:* allelic richness (mean of all loci); *H*_*O*_: observed heterozygosity averaged over loci; *H*_*E*_: average expected heterozygosity; *L*_*HWD*_: number of loci which significantly deviate from HWE at significance level 0.01 or 0.05 in brackets (also see Supplementary Table S2); *N*_*mt*_: number of individuals with mtDNA sequence data; *N*_*H*_: total number of mitochondrial haplotypes; *N*_*pH*_ and *N*_*p%*_: total number and percentage of private haplotypes; *H*_*d*_: haplotype diversity; *π:* nucleotide diversity.

## References

[b1] Avise J. C. Phylogeography: retrospect and prospect. J. Biogeogr. 36, 3–15 (2009).

[b2] Hewitt G. M. Quaternary phylogeography the roots of hybrid zones. Genetica 139, 617–638 (2011). 21234647 10.1007/s10709-011-9547-3

[b3] Abbott R. . Hybridization and speciation. J. Evol. Biol. 26, 229–246 (2013). 23323997 10.1111/j.1420-9101.2012.02599.x

[b4] Hewitt G. M. Post-glacial re-colonization of European biota. Biol. J. Linn. Soc. 68, 87–112 (1999).

[b5] Schmitt T. Molecular biogeography of Europe: Pleistocene cycles and postglacial trends. Frontiers Zool. 4, 11 (2007). 10.1186/1742-9994-4-11 PMC1868914 17439649

[b6] Gompert Z. . Admixture and the organization of genetic diversity in a butterfly species complex revealed through common and rare genetic variants. Mol. Ecol. 23, 4555–4573 (2014). 24866941 10.1111/mec.12811

[b7] Buggs R. J. A. Empirical study of hybrid zone movement. Heredity 99, 301–312 (2007). 17611495 10.1038/sj.hdy.6800997

[b8] Scriber J. M. Impacts of climate warming on hybrid zone movement: geographically diffuse and biologically porous “species borders”. Insect Sci. 18, 121–159 (2011).

[b9] Canestrelli D., Bisconti R. & Nascetti G. Extensive unidirectional introgression between two salamander lineages of ancient divergence and its evolutionary implications. Sci. Rep. 4, 6516 (2014). 25269625 10.1038/srep06516 PMC5377473

[b10] Dapporto L. & Bruschini C. Invading a refugium: post glacial replacement of the ancestral lineage of a nymphalid butterfly in the West Mediterranean. Org. Divers. Evol. 12, 39–49 (2012).

[b11] Mallet J., Wynne I. R. & Thomas C. D. Hybridisation and climate change: brown argus butterflies in Britain (*Polyommatus* subgenus Aricia). Insect Cons. Div. 4, 192–199 (2010).

[b12] Mende M. B. & Hundsdoerfer A. K. Mitochondrial lineage sorting in action – historical biogeography of the *Hyles euphorbiae* complex (Sphingidae, Lepidoptera) in Italy. BMC Evol. Biol. 13, 83 (2013). 23594258 10.1186/1471-2148-13-83 PMC3655913

[b13] Mende M. B. & Hundsdoerfer A. K. More evidence for an admixture of the *Hyles euphorbiae* complex’s main lineages in Mediterranean Europe (Lepidoptera: Sphingidae). Eur. J. Entomol. 111, 584–587 (2014).

[b14] Hundsdoerfer A. K., Kitching I. J. & Wink M. A molecular phylogeny of the hawkmoth genus *Hyles* (Lepidoptera: Sphingidae, Macroglossinae). Mol. Phylogen. Evol. 35, 442–458 (2005). 10.1016/j.ympev.2005.02.004 15804414

[b15] Hundsdoerfer A. K., Kitching I. J. & Wink M. The phylogeny of the *Hyles euphorbiae*-complex (Lepidoptera: Sphingidae): molecular evidence from sequence data and ISSR-PCR fingerprints. Org. Divers. Evol. 5, 173–198 (2005).

[b16] Hundsdoerfer A. K., Mende M. B., Kitching I. J. & Cordellier M. Taxonomy, phylogeography and climate relations of the Western Palaearctic spurge hawkmoth (Lepidoptera, Sphingidae, Macroglossinae). Zool. Scr. 40, 403–417 (2011).

[b17] Pittaway A. R. The Hawkmoths of the Western Palaearctic. 240 (Harley Books, Colchester, UK, 1993).

[b18] Hundsdoerfer A. K., Rubinoff D., Attié M., Kitching I. J. & Wink M. A revised molecular phylogeny of the globally distributed hawkmoth genus *Hyles* (Lepidoptera: Sphingidae), based on mitochondrial and nuclear DNA sequences. Mol. Phylogen. Evol. 52, 852–865 (2009). 10.1016/j.ympev.2009.05.023 19482093

[b19] Kitching I. J. *Sphingidae Taxonomic Inventory.* Available at: http://sphingidae.myspecies.info/(Accessed: 5th October 2015) (2015).

[b20] Hundsdoerfer A. K., Mende M. B., Harbich H., Pittaway A. R. & Kitching I. J. Larval pattern morphotypes in the Western Palaearctic *Hyles euphorbiae* complex (Lepidoptera: Sphingidae: Macroglossinae). Insect Syst. Phyl. 42, 41–86 (2011).

[b21] Danner F., Eitschberger U. & Surholt B. Die Schwärmer der westlichen Palaearktis. Bausteine zu einer Revision (Lepidoptera: Sphingidae). Herbipoliana 4, 1–368 (1998).

[b22] Harbich H. Der *Hyles euphorbiae*-Komplex–ein taxonomisches Problem? (Lepidoptera: Sphingidae) 6. Teil. Entomol. Z. 104, 61–84 (1994).

[b23] Harbich H. Anmerkungen zur Wolfsmilchschwärmerpopulation (*Hyles euphorbiae* (Linnaeus, 1758)-Komplex) von Malta (Lepidoptera: Sphingidae). Entomol. Z. 119, 51–58 (2009).

[b24] Funk D. J. & Omland K. E. Species-level paraphyly and polyphyly: Frequency, causes, and consequences, with insights from animal mitochondrial DNA. Ann. Rev. Ecol. Evol. System. 34, 397–423 (2003).

[b25] Flanders J. . Phylogeography of the greater horseshoe bat, *Rhinolophus ferrumequinum*: contrasting results from mitochondrial and microsatellite data. Mol. Ecol. 18, 306–318 (2009). 19192181 10.1111/j.1365-294X.2008.04021.x

[b26] Mallet J. Hybridization as an invasion of the genome. Trends Ecol. Evol. 20, 229–237 (2005). 16701374 10.1016/j.tree.2005.02.010

[b27] Toews D. P. L. & Brelsford A. The biogeography of mitochondrial and nuclear discordance in animals. Mol. Ecol. 21, 3907–3930 (2012). 22738314 10.1111/j.1365-294X.2012.05664.x

[b28] Hall T. A. BioEdit: a user-friendly biological sequence alignment editor and analysis program for Windows 95/98/NT. Nucleic Acids Symp. Ser. 41, 95–98 (1999).

[b29] Mende M. B., Stuckas H. & Hundsdoerfer A. K. Eight new microsatellite loci of the Western Palearctic *Hyles euphorbiae* complex (Lepidoptera, Sphingidae). Ann. Zool. Fenn. 48, 142–146 (2011).

[b30] Hundsdoerfer A. K., Sanetra M., Corbeil D. & Stuckas H. Eleven hawkmoth microsatellite loci of Canary Island *Hyles tithymali* (Lepidoptera). Conserv. Gen. Res. 2, 241–244 (2009).

[b31] Tamura K., Dudley J., Nei M. & Kumar S. MEGA4: Molecular Evolutionary Genetics Analysis (MEGA) software version 4.0. Mol. Biol. Evol. 24, 1596–1599 (2007). 17488738 10.1093/molbev/msm092

[b32] Librado P. & Rozas J. DNASP v5: a software for comprehensive analysis of DNA polymorphism data. Bioinformatics 25, 1451–1452 (2009). 19346325 10.1093/bioinformatics/btp187

[b33] Excoffier L., Laval G. & Schneider S. ARLEQUIN (version 3.0): an integrated software package for population genetics data analysis. Evol. Bioinform. Online 1, 47–50 (2005). 19325852 PMC2658868

[b34] Drummond A. J. & Rambaut A. BEAST: Bayesian evolutionary analysis by sampling trees. BMC Evol. Biol. 7, 214 (2007). 17996036 10.1186/1471-2148-7-214 PMC2247476

[b35] Drummond A. J., Suchard M. A., Xie D. & Rambaut A. Bayesian phylogenetics with BEAUti and the BEAST 1.7. Mol. Biol. Evol. 29, 1969–1973 (2012). 22367748 10.1093/molbev/mss075 PMC3408070

[b36] Papadopoulou A., Anastasiou I. & Vogler A. P. Revisiting the insect mitochondrial molecular clock: The mid-Aegean trench calibration. Mol. Biol. Evol. 27, 1659–1672 (2010). 20167609 10.1093/molbev/msq051

[b37] Schenekar T. & Weiss S. High rate of calculation errors in mismatch distribution analysis results in numerous false inferences of biological importance. Heredity 107, 511–512 (2011). 21731052 10.1038/hdy.2011.48 PMC3242633

[b38] Rambaut A., Suchard M. & Drummond A. J. *Tracer v1.0-1.6.* Available at: http://tree.bio.ed.ac.uk/sofware/tracer/ (Accessed: 19th September 2014) (2015).

[b39] Rousset F. GENEPOP’007: a complete reimplementation of the GENEPOP software for Windows and Linux. Mol. Ecol. Res. 8, 103–106 (2008). 10.1111/j.1471-8286.2007.01931.x 21585727

[b40] van Oosterhout C., Hutchinson W. F., Wills D. P. M. & Shipley P. MICRO-CHECKER: software for identifying and correcting genotyping errors in microsatellite data. Mol. Ecol. Notes 4, 535–538 (2004).

[b41] Jensen J. L., Bohonak A. J. & Kelley S. T. Isolation by distance, web service. BMC Genet. 6, 13 (2005). 15760479 10.1186/1471-2156-6-13 PMC1079815

[b42] Ersts P. J. *Geographic Distance Matrix Generator (version 1.2.3).* Available at: http://biodiversityinformatics.amnh.org/open_source/gdmg (Accessed: 10th November 2009) (2007).

[b43] Rosenberg M. S. & Anderson C. D. PASSaGE: Pattern Analysis, Spatial Statistics and Geographic Exegesis. Version 2. Methods in Ecology & Evolution 2, 229–232 (2011).

[b44] Pritchard J. K., Stephens M. & Donnelly P. Inference of population structure using multilocus genotype data. Genetics 155, 945–959 (2000). 10835412 10.1093/genetics/155.2.945 PMC1461096

[b45] Guillot G., Estoup A., Mortier F. & Cosson J. F. A spatial statistical model for landscape genetics. Genetics 170, 1261–1280 (2005). 15520263 10.1534/genetics.104.033803 PMC1451194

[b46] Guillot G., Mortier F. & Estoup A. Geneland: A computer package for landscape genetics. Mol. Ecol. Notes 5, 708–711 (2005).

[b47] Falush D., Stephens M. & Pritchard J. K. Inference of population structure using multilocus genotype data: dominant markers and null alleles. Mol. Ecol. Notes 7, 574–578 (2007). 18784791 10.1111/j.1471-8286.2007.01758.x PMC1974779

[b48] Guillot G., Santos F. & Estoup A. Analysing georeferenced population genetics data with Geneland: a new algorithm to deal with null alleles and a friendly graphical user interface. Bioinformatics 24, 1406–1407 (2008). 18413327 10.1093/bioinformatics/btn136

[b49] Falush D., Stephens M. & Pritchard J. K. Inference of population structure using multilocus genotype data: linked loci and correlated allele frequencies. Genetics 164, 1567–1587 (2003). 12930761 10.1093/genetics/164.4.1567 PMC1462648

[b50] Evanno G., Regnaut S. & Goudet J. Detecting the number of clusters of individuals using the software STRUCTURE: a simulation study. Mol. Ecol. 14, 2611–2620 (2005). 15969739 10.1111/j.1365-294X.2005.02553.x

[b51] Rosenberg N. A. DISTRUCT: a program for the graphical display of population structure. Mol. Ecol. Notes 4, 137–138 (2004).

[b52] Fontaine M. C. . Rise of oceanographic barriers in continuous populations of a cetacean: the genetic structure of harbour porpoises in Old World waters. BMC Biol. 5, 30 (2007). 17651495 10.1186/1741-7007-5-30 PMC1971045

[b53] Jombart T. Adegenet: a R package for the multivariate analysis of genetic markers. Bioinformatics 24, 1403–1405 (2008). 18397895 10.1093/bioinformatics/btn129

[b54] Goudet J. FSTAT (Version 1.2): A computer program to calculate F-statistics. J. Hered. 86, 485–486 (1995).

[b55] Glaubitz J. C. CONVERT: a user friendly program to reformat diploid genotypic data for commonly used population genetic software packages. Mol. Ecol. Notes 4, 309–310 (2004).

[b56] Heller R., Chikhi L. & Siegismund H. R. The confounding effect of population structure on Bayesian skyline plot inferences of demographic history. PLoS One 8, e62992 (2013). 23667558 10.1371/journal.pone.0062992 PMC3646956

[b57] Husemann M., Schmitt T., Zachos F. E., Ulrich W. & Habel J. C. Palaearctic biogeography revisited: evidence for the existence of a North African refugium for Western Palaearctic biota. J. Biogeogr. 41, 81–94 (2014).

[b58] Waters J. M., Fraser C. I. & Hewitt G. M. Founder takes all: density-dependent processes structure biodiversity. Trends Ecol. Evol. 28, 78–85 (2013). 23000431 10.1016/j.tree.2012.08.024

[b59] Petit R. J. Early insights into the genetic consequences of range expansions. Heredity 106, 203–204 (2011). 20502481 10.1038/hdy.2010.60 PMC3183874

[b60] Adams J. M. & Faure H. *Review and Atlas of Palaeovegetation: Preliminary land ecosystem maps of the world since the Last Glacial Maximum*. Available at: http://www.esd.ornl.gov/ern/qen/adams1.html (Accessed: August 2014) (1997).

[b61] Küster H. Geschichte der Landschaft in Mitteleuropa: von der Eiszeit bis zur Gegenwart. (CH Beck, 2010).

[b62] Carcasson R. H. Revised catalogue of the African Sphingidae (Lepidoptera) with descriptions of the East African species. J. East Afr. Nat. Hist. 26, 1–148 (1968).

[b63] Jolly D. . Biome reconstruction from pollen and plant macrofossil data for Africa and the Arabian peninsula at 0 and 6000 years. J. Biogeogr. 25, 1007–1027 (1998).

[b64] Meirmans P. G. The trouble with isolation by distance. Mol. Ecol. 21, 2839–2846 (2012). 22574758 10.1111/j.1365-294X.2012.05578.x

[b65] Schwartz M. K. & McKelvey K. S. Why sampling scheme matters: the effect of sampling scheme on landscape genetic results. Conserv. Genet. 10, 441–452 (2009).

[b66] Diniz-Filho J. A. F. . Mantel test in population genetics. Genet. Mol. Biol. 36, 475–485 (2013). 24385847 10.1590/S1415-47572013000400002 PMC3873175

[b67] Hermansen J. S. . Hybrid speciation in sparrows I: phenotypic intermediacy, genetic admixture and barriers to gene flow. Mol. Ecol. 20, 3812–3822 (2011). 21771138 10.1111/j.1365-294X.2011.05183.x

[b68] Gil-T F., Requejo S. & Estevez R. A new relict species [sic] for the Iberian Peninsula, with an enigmatic distribution: *Hyles tithymali gallaeci* subspec. nov. from the Atlantic islands and coast of Galicia Region (NW-Spain). Atalanta 42, 143–148 (2011).

[b69] Poelstra J. W. . The genomic landscape underlying phenotypic integrity in the face of gene flow in crows. Science 344, 1410–1414 (2014). 24948738 10.1126/science.1253226

[b70] Harbich H. & Hundsdoerfer A. K. Untersuchungen an einem *Hyles* Freilandhybriden aus Nord Tunesien (Lepidoptera: Sphingidae). Entomol. Z. 116, 99–106 (2006).

[b71] Currat M., Ruedi M., Petit R. J. & Excoffier L. The hidden side of invasions: Massive introgression by local genes. Evolution 62, 1908–1920 (2008). 18452573 10.1111/j.1558-5646.2008.00413.x

[b72] Werren J. H., Baldo L. & Clark M. E. Wolbachia: master manipulators of invertebrate biology. Nature Rev. Microbiol. 6, 741–751 (2008). 18794912 10.1038/nrmicro1969

[b73] Ballard J. W. O. & Melvin R. G. Linking the mitochondrial genotype to the organismal phenotype. Mol. Ecol. 19, 1523–1539 (2010). 20345689 10.1111/j.1365-294X.2010.04594.x

[b74] Sun J.-T. . Evidence for high dispersal ability and mito-nuclear discordance in the small brown planthopper, Laodelphax striatellus. Sci. Rep. 5, 8045 (2015). 25622966 10.1038/srep08045 PMC4309506

[b75] Irwin D. E. Local adaptation along smooth ecological gradients causes phylogeographic breaks and phenotypic clustering. The Am. Nat. 180, 35–49 (2012). 22673649 10.1086/666002

[b76] Davis B. A. S., Brewer S., Stevenson A. C., Guiot J. & Data Contributors. The temperature of Europe during the Holocene reconstructed from pollen data. Quatern. Sci. Rev. 22, 1701–1716 (2003).

[b77] Garcia-Mudarra J. L., Ibanez C. & Juste J. The Straits of Gibraltar: barrier or bridge to Ibero-Moroccan bat diversity? Biol. J. Linn. Soc. 96, 434–450 (2009).

[b78] Brantjes N. B. M. Wind as a factor influencing flower-visiting by H*adena bicruris* (Noctuidae) and *Deilephila elpenor* (Sphingidae). Ecol. Entomol. 6, 361–363 (1981).

[b79] Catania A. *Hyles tithymali deserticola* (Staudinger, 1901)–first record for the Maltese Islands (Lepidoptera: Sphingidae). SHILAP Rev. Lepidopterol. 36, 69–71 (2008).

[b80] Hajibabaei M., Singer G. A. C., Hebert P. D. N. & Hickey D. A. DNA barcoding: how it complements taxonomy, molecular phylogenetics and population genetics. Trends Ecol. Evol. 23, 167–172 (2007). 10.1016/j.tig.2007.02.001 17316886

[b81] Randi E. Detecting hybridization between wild species and their domesticated relatives. Mol. Ecol. 17, 285–293 (2008). 18173502 10.1111/j.1365-294X.2007.03417.x

