# Supplementary material for: A comprehensive phylogeography of the Hyles euphorbiae complex (Lepidoptera: Sphingidae) indicates a ‘glacial refuge belt’
Source: Sci Rep. 2016 Jul 21;6:29527. doi: 10.1038/srep29527 (PMC4954964; doi:10.1038/srep29527)
Supplement: Supplementary Information [file srep29527-s1.doc]

**A comprehensive Phylogeography of the *Hyles euphorbiae* complex (Lepidoptera: Sphingidae) indicates a** '**glacial refuge belt**'

Michael B. Mende^1, 2, 3^,*

Manuela Bartel^1^

Anna K. Hundsdoerfer^1, 2^

**Supplementary information**

**Supplementary tables**

**Table S1.** List of samples, corresponding data and microsatellite alleles table.

*MTD-TW #:* voucher number in the ‘Museum für Tierkunde’, Dresden (invertebrate tissue collection); *Ind. #:* individual collection number; *italic coordinates* estimated from locality name; *Taxon:* traditionally defined taxon affiliation; *HT#:* mitochondrial haplogroup assignment (A = '*euphorbiae*', B = '*tithymali*', C = '*melitensis*', D = '*italica*', E = '*cretica*', F = '*enigmatica*', G = '*robertsi*', H = '*dahlii*') and haplotype number; *GB Acc.#:* GenBank accession number for mtDNA sequence, individuals without sequence data indicated by “n/a”; allele length data is given for the 12 microsatellite loci in the following columns; individuals without microsatellite data indicated by “n/a” in the first column. Individuals pooled to populations according to Fig. 1 with abbreviation (* = putative hybrid population), number of individuals (*n*) and average coordinates given. Mitochondrial data of the Saudi Arabian population was not included in computational analyses because only museum specimens were available for which we sequenced only three short fragments for haplogroup assignment according to protocols of Mende & Hundsdoerfer^1^.

See “Supplementary Information” (Excel file) for Supplementary Table S1

**Table S2.** Characteristics of 12 microsatellite loci.

*Locus:* name of microsatellite locus, ^2^ = described in Hundsdoerfer *et al.*^2^, ^3^ = described in Mende *et al.*^3^, F_ST_ = differentiation of *K*=2 Structure clusters at the respective locus with insignificant values (*p* < 0.05) indicated in italic; *Pop:* population abbreviations correspond to Fig. 1 (* = putative hybrid populations; only populations with <15 samples); *N:* number of individuals; *H_O_:* observed heterozygosity; *H_E_:* expected heterozygosity; *P_HWE_:* probability that population is in HWE, *mono.* = monomorphic, ** = null allele bias.

| **Locus** | **Pop** | ***N*** | **H_O_** | **H_E_** | **P_HWE_** | |  | **Locus** | **Pop** | ***N*** | **H_O_** | **H_E_** | **P_HWE_** | |
| --- | --- | --- | --- | --- | --- | --- | --- | --- | --- | --- | --- | --- | --- | --- |
| Hyti13^2^ | IRA | 31 | 0.7742 | 0.8429 | 0.2568 |  |  | Hyti22^2^ | IRA | 31 | 0.0645 | 0.4865 | < 0.00015 | ** |
| F_ST_=0.047 | BUL | 33 | 0.8182 | 0.8303 | 0.0482 |  |  | F_ST_=*0.001* | BUL | 33 | 0.3333 | 0.4904 | 0.0878 |  |
|  | SGR | 17 | 0.7647 | 0.7986 | 0.4072 |  |  |  | SGR | 17 | 0.2353 | 0.5829 | 0.0057 | ** |
|  | *CRE | 16 | 0.6875 | 0.7903 | 0.0498 |  |  |  | *CRE | 16 | 0.1875 | 0.2802 | 0.2997 |  |
|  | HUN | 24 | 0.7917 | 0.8112 | 0.7681 |  |  |  | HUN | 24 | 0.3333 | 0.5106 | 0.1143 |  |
|  | SLK | 45 | 0.9091 | 0.8393 | 0.0368 |  |  |  | SLK | 45 | 0.2444 | 0.4484 | < 0.00076 | ** |
|  | GER | 33 | 0.7576 | 0.8252 | 0.0260 |  |  |  | GER | 33 | 0.2813 | 0.4737 | 0.0082 | ** |
|  | BEL | 18 | 0.7778 | 0.7778 | 0.4417 |  |  |  | BEL | 18 | 0.2778 | 0.5000 | 0.1391 |  |
|  | ESP | 26 | 0.8077 | 0.8017 | 0.5442 |  |  |  | ESP | 26 | 0.3846 | 0.5332 | 0.1691 |  |
|  | SSP | 20 | 0.8000 | 0.7077 | 0.9421 |  |  |  | SSP | 20 | 0.2500 | 0.4987 | 0.0139 | ** |
|  | NIT | 33 | 0.8750 | 0.8829 | 0.3315 |  |  |  | NIT | 33 | 0.3226 | 0.5135 | 0.0574 | ** |
|  | *EIT | 25 | 0.6800 | 0.8539 | 0.0377 | ** |  |  | *EIT | 25 | 0.4167 | 0.4787 | 0.5276 |  |
|  | *CIT | 45 | 0.8222 | 0.7933 | 0.9889 |  |  |  | *CIT | 45 | 0.3182 | 0.5016 | 0.0168 |  |
|  | *SIT | 28 | 0.7857 | 0.7877 | 0.4860 |  |  |  | *SIT | 28 | 0.2857 | 0.5253 | 0.0129 | ** |
|  | *SIC | 23 | 0.6087 | 0.7710 | 0.0450 |  |  |  | *SIC | 23 | 0.1304 | 0.5034 | < 0.00015 | ** |
|  | *PAN | 41 | 0.7317 | 0.7016 | 0.5595 |  |  |  | *PAN | 41 | 0.4500 | 0.4608 | 1.0000 |  |
|  | *MAL | 29 | 0.8276 | 0.6872 | 0.8267 |  |  |  | *MAL | 29 | 0.1035 | 0.2160 | 0.0305 |  |
|  | *TUN | 22 | 0.5455 | 0.5211 | 0.6327 |  |  |  | *TUN | 22 | 0.1364 | 0.2738 | 0.0560 |  |
|  | *MOR | 20 | 0.8500 | 0.7641 | 0.1661 |  |  |  | *MOR | 20 | 0.4000 | 0.4308 | 1.0000 |  |
|  | cFV | 24 | 0.7917 | 0.6605 | 0.2082 |  |  |  | cFV | 24 | 0.3750 | 0.4885 | 0.3899 |  |
|  | cGC | 80 | 0.3750 | 0.5564 | < 0.00076 | ** |  |  | cGC | 80 | 0.2875 | 0.4993 | < 0.00076 | ** |
|  | cTF | 32 | 0.5938 | 0.5650 | 0.1582 |  |  |  | cTF | 32 | 0.2188 | 0.4836 | 0.0028 | ** |
|  | cLG | 19 | 0.7895 | 0.6899 | 0.4155 |  |  |  | cLG | 19 | 0.2632 | 0.4623 | 0.1152 |  |
|  | cEH | 29 | 0.3214 | 0.5734 | 0.0062 | ** |  |  | cEH | 29 | 0.2069 | 0.3727 | 0.0290 | ** |
|  | cLP | 31 | 0.4839 | 0.6499 | 0.1837 |  |  |  | cLP | 31 | 0.2258 | 0.5124 | < 0.00076 | ** |
|  | FOG | 23 | 0.7826 | 0.6251 | 0.4920 |  |  |  | FOG | 23 | 0.1739 | 0.1623 | 1.0000 |  |
| Hyti14^2^ | IRA | 31 | 0.3226 | 0.3231 | 1.0000 |  |  | Hyti41^2^ | IRA | 31 | 0.7742 | 0.9122 | 0.0134 | ** |
| F_ST_=*0.003* | BUL | 33 | 0.2727 | 0.4713 | 0.0159 | ** |  | F_ST_=0.038 | BUL | 33 | 0.8485 | 0.9203 | 0.0050 |  |
|  | SGR | 17 | 0.0000 | 0.0000 | *mono.* |  |  |  | SGR | 17 | 0.7647 | 0.9305 | 0.0441 |  |
|  | *CRE | 16 | 0.1250 | 0.1210 | 1.0000 |  |  |  | *CRE | 16 | 0.8750 | 0.8347 | 0.9373 |  |
|  | HUN | 24 | 0.2500 | 0.2943 | 0.5034 |  |  |  | HUN | 24 | 0.7083 | 0.8936 | 0.1281 | ** |
|  | SLK | 45 | 0.4222 | 0.4127 | 0.8852 |  |  |  | SLK | 45 | 0.8222 | 0.9253 | < 0.00015 | ** |
|  | GER | 33 | 0.2813 | 0.2495 | 1.0000 |  |  |  | GER | 33 | 0.7273 | 0.8793 | < 0.00076 | ** |
|  | BEL | 18 | 0.0000 | 0.0000 | *mono.* |  |  |  | BEL | 18 | 0.7222 | 0.8635 | 0.1517 |  |
|  | ESP | 26 | 0.1539 | 0.2451 | 0.0575 |  |  |  | ESP | 26 | 0.8077 | 0.9012 | 0.3358 |  |
|  | SSP | 20 | 0.0000 | 0.0000 | *mono.* |  |  |  | SSP | 20 | 0.8000 | 0.8218 | 0.0015 |  |
|  | NIT | 33 | 0.3333 | 0.4490 | 0.1073 |  |  |  | NIT | 33 | 0.7273 | 0.8904 | 0.0053 | ** |
|  | *EIT | 25 | 0.3600 | 0.3078 | 1.0000 |  |  |  | *EIT | 25 | 0.7600 | 0.8629 | 0.0083 |  |
|  | *CIT | 45 | 0.0444 | 0.0442 | 1.0000 |  |  |  | *CIT | 45 | 0.6667 | 0.9084 | < 0.00015 | ** |
|  | *SIT | 28 | 0.1429 | 0.2591 | 0.0106 | ** |  |  | *SIT | 28 | 0.7778 | 0.9154 | 0.1198 | ** |
|  | *SIC | 23 | 0.0435 | 0.0435 | 1.0000 |  |  |  | *SIC | 23 | 0.6522 | 0.9304 | < 0.00015 | ** |
|  | *PAN | 41 | 0.0000 | 0.0000 | *mono.* |  |  |  | *PAN | 41 | 0.7317 | 0.8308 | 0.2709 |  |
|  | *MAL | 29 | 0.0370 | 0.1069 | 0.0561 |  |  |  | *MAL | 29 | 0.7586 | 0.8724 | 0.0290 |  |
|  | *TUN | 22 | 0.1429 | 0.1382 | 1.0000 |  |  |  | *TUN | 22 | 0.4546 | 0.6311 | < 0.00015 | ** |
|  | *MOR | 20 | 0.2000 | 0.1897 | 1.0000 |  |  |  | *MOR | 20 | 0.7368 | 0.8506 | 0.5228 |  |
|  | cFV | 24 | 0.3333 | 0.3927 | 0.6604 |  |  |  | cFV | 24 | 0.6250 | 0.8936 | 0.0093 | ** |
|  | cGC | 80 | 0.3750 | 0.4452 | 0.0484 |  |  |  | cGC | 80 | 0.4875 | 0.8491 | < 0.00015 | ** |
|  | cTF | 32 | 0.1290 | 0.2322 | 0.0504 |  |  |  | cTF | 32 | 0.7813 | 0.8547 | 0.1083 |  |
|  | cLG | 19 | 0.1579 | 0.2347 | 0.2610 |  |  |  | cLG | 19 | 0.7368 | 0.7681 | 0.5501 |  |
|  | cEH | 29 | 0.1724 | 0.1603 | 1.0000 |  |  |  | cEH | 29 | 0.6296 | 0.8896 | 0.0036 | ** |
|  | cLP | 31 | 0.1290 | 0.1227 | 1.0000 |  |  |  | cLP | 31 | 0.8387 | 0.8578 | 0.9915 |  |
|  | FOG | 23 | 0.1739 | 0.2937 | 0.0981 |  |  |  | FOG | 23 | 0.3478 | 0.7179 | < 0.00076 | ** |
|  |  |  |  |  |  |  |  |  |  |  |  |  |  |  |
| **Locus** | **Pop** | ***N*** | **H_O_** | **H_E_** | **P_HWE_** |  |  | **Locus** | **Pop** | ***N*** | **H_O_** | **H_E_** | **P_HWE_** |  |
| Hyti49^2^ | IRA | 31 | 0.2333 | 0.8316 | < 0.00015 | ** |  | Hti62^3^ | IRA | 31 | 0.2000 | 0.9388 | < 0.00015 | ** |
| F_ST_=0.028 | BUL | 33 | 0.1212 | 0.8023 | < 0.00015 | ** |  | F_ST_=0.091 | BUL | 33 | 0.2069 | 0.8960 | < 0.00015 | ** |
|  | SGR | 17 | 0.4118 | 0.8378 | < 0.00015 | ** |  |  | SGR | 17 | 0.4286 | 0.8915 | < 0.00015 | ** |
|  | *CRE | 16 | 0.2500 | 0.7016 | < 0.00015 | ** |  |  | *CRE | 16 | 0.1667 | 0.4394 | 0.0898 |  |
|  | HUN | 24 | 0.3044 | 0.8715 | < 0.00015 | ** |  |  | HUN | 24 | 0.4500 | 0.9436 | < 0.00015 | ** |
|  | SLK | 45 | 0.4444 | 0.7810 | < 0.00015 | ** |  |  | SLK | 45 | 0.5333 | 0.9229 | < 0.00015 | ** |
|  | GER | 33 | 0.1724 | 0.8179 | < 0.00015 | ** |  |  | GER | 33 | 0.3226 | 0.9043 | < 0.00015 | ** |
|  | BEL | 18 | 0.2353 | 0.7005 | < 0.00015 | ** |  |  | BEL | 18 | 0.5000 | 0.7841 | < 0.00076 | ** |
|  | ESP | 26 | 0.2083 | 0.8200 | < 0.00015 | ** |  |  | ESP | 26 | 0.3000 | 0.9346 | < 0.00015 | ** |
|  | SSP | 20 | 0.2000 | 0.6513 | < 0.00015 | ** |  |  | SSP | 20 | 0.4444 | 0.8413 | < 0.00015 | ** |
|  | NIT | 33 | 0.2500 | 0.8492 | < 0.00015 | ** |  |  | NIT | 33 | 0.6207 | 0.9371 | < 0.00015 | ** |
|  | *EIT | 25 | 0.5000 | 0.8192 | 0.0010 | ** |  |  | *EIT | 25 | 0.2083 | 0.7367 | < 0.00015 | ** |
|  | *CIT | 45 | 0.5000 | 0.8294 | < 0.00015 | ** |  |  | *CIT | 45 | 0.4048 | 0.9131 | < 0.00015 | ** |
|  | *SIT | 28 | 0.3571 | 0.8351 | < 0.00015 | ** |  |  | *SIT | 28 | 0.4800 | 0.9037 | < 0.00015 | ** |
|  | *SIC | 23 | 0.5217 | 0.7324 | 0.0126 | ** |  |  | *SIC | 23 | 0.4286 | 0.8444 | < 0.00015 | ** |
|  | *PAN | 41 | 0.2683 | 0.5014 | < 0.00015 | ** |  |  | *PAN | 41 | 0,0000 | 0.7344 | < 0.00015 | ** |
|  | *MAL | 29 | 0.1724 | 0.3388 | 0.0095 | ** |  |  | *MAL | 29 | 0.1429 | 0.6533 | < 0.00015 | ** |
|  | *TUN | 22 | 0.0909 | 0.3256 | < 0.00015 | ** |  |  | *TUN | 22 | 0.8182 | 0.7696 | 0.0347 |  |
|  | *MOR | 20 | 0.3684 | 0.8265 | < 0.00076 | ** |  |  | *MOR | 20 | 0.3125 | 0.8327 | < 0.00015 | ** |
|  | cFV | 24 | 0.4167 | 0.8502 | < 0.00015 | ** |  |  | cFV | 24 | 0.5000 | 0.6551 | 0.0473 |  |
|  | cGC | 80 | 0.2125 | 0.8577 | < 0.00015 | ** |  |  | cGC | 80 | 0.4935 | 0.6297 | < 0.00015 | ** |
|  | cTF | 32 | 0.2903 | 0.7657 | < 0.00015 | ** |  |  | cTF | 32 | 0.4839 | 0.6076 | 0.0482 |  |
|  | cLG | 19 | 0.3529 | 0.7932 | < 0.00076 | ** |  |  | cLG | 19 | 0.4737 | 0.6117 | 0.0963 |  |
|  | cEH | 29 | 0.2143 | 0.8084 | < 0.00015 | ** |  |  | cEH | 29 | 0.5862 | 0.6267 | 0.0236 |  |
|  | cLP | 31 | 0.3226 | 0.8699 | < 0.00015 | ** |  |  | cLP | 31 | 0.5667 | 0.6633 | 0.1813 |  |
|  | FOG | 23 | 0.2174 | 0.7604 | < 0.00015 | ** |  |  | FOG | 23 | 0.0870 | 0.5961 | < 0.00015 | ** |
| Hyti50^2^ | IRA | 31 | 0.0645 | 0.0635 | 1.0000 |  |  | Hti63^3^ | IRA | 31 | 0.0323 | 0.0323 | 1.0000 |  |
| F_ST_=0.377 | BUL | 33 | 0.1818 | 0.1702 | 1.0000 |  |  | F_ST_=0.393 | BUL | 33 | 0.0000 | 0.0000 | *mono.* |  |
|  | SGR | 17 | 0.2941 | 0.2585 | 1.0000 |  |  |  | SGR | 17 | 0.1765 | 0.1658 | 1.0000 |  |
|  | *CRE | 16 | 0.1875 | 0.2319 | 0.1928 |  |  |  | *CRE | 16 | 0.0000 | 0.0000 | *mono.* |  |
|  | HUN | 24 | 0.0833 | 0.0825 | 1.0000 |  |  |  | HUN | 24 | 0.1250 | 0.1215 | 1.0000 |  |
|  | SLK | 45 | 0.0444 | 0.0442 | 1.0000 |  |  |  | SLK | 45 | 0.0444 | 0.0440 | 1.0000 |  |
|  | GER | 33 | 0.3030 | 0.3380 | 0.3681 |  |  |  | GER | 33 | 0.0000 | 0.0000 | *mono.* |  |
|  | BEL | 18 | 0.0000 | 0.0000 | *mono.* |  |  |  | BEL | 18 | 0.0000 | 0.0000 | *mono.* |  |
|  | ESP | 26 | 0.0385 | 0.0385 | 1.0000 |  |  |  | ESP | 26 | 0.1154 | 0.1471 | 0.1214 |  |
|  | SSP | 20 | 0,0000 | 0.2615 | < 0.00076 | ** |  |  | SSP | 20 | 0.1000 | 0.0974 | 1.0000 |  |
|  | NIT | 33 | 0.0303 | 0.1441 | 0.0008 | ** |  |  | NIT | 33 | 0.1818 | 0.1702 | 1.0000 |  |
|  | *EIT | 25 | 0.3200 | 0.2743 | 1.0000 |  |  |  | *EIT | 25 | 0.0800 | 0.0784 | 1.0000 |  |
|  | *CIT | 45 | 0.1333 | 0.1258 | 1.0000 |  |  |  | *CIT | 45 | 0.1111 | 0.1061 | 1.0000 |  |
|  | *SIT | 28 | 0.0741 | 0.0727 | 1.0000 |  |  |  | *SIT | 28 | 0.2500 | 0.2747 | 0.5318 |  |
|  | *SIC | 23 | 0.1739 | 0.1623 | 1.0000 |  |  |  | *SIC | 23 | 0.5652 | 0.4493 | 0.3445 |  |
|  | *PAN | 41 | 0.0000 | 0.0000 | *mono.* |  |  |  | *PAN | 41 | 0.1951 | 0.1783 | 1.0000 |  |
|  | *MAL | 29 | 0.1724 | 0.1603 | 1.0000 |  |  |  | *MAL | 29 | 0.7241 | 0.5003 | 0.0158 |  |
|  | *TUN | 22 | 0.0909 | 0.0888 | 1.0000 |  |  |  | *TUN | 22 | 0.1364 | 0.3837 | 0.0068 | ** |
|  | *MOR | 20 | 0.4000 | 0.4308 | 1.0000 |  |  |  | *MOR | 20 | 0.2632 | 0.4623 | 0.1166 |  |
|  | cFV | 24 | 0.7083 | 0.4885 | 0.0356 |  |  |  | cFV | 24 | 0.3750 | 0.4672 | 0.3924 |  |
|  | cGC | 80 | 0.4625 | 0.4899 | 0.6490 |  |  |  | cGC | 80 | 0.5769 | 0.5138 | 0.6282 |  |
|  | cTF | 32 | 0.4688 | 0.4678 | 1.0000 |  |  |  | cTF | 32 | 0.5313 | 0.5258 | 1.0000 |  |
|  | cLG | 19 | 0.3333 | 0.4571 | 0.3105 |  |  |  | cLG | 19 | 0.6842 | 0.5107 | 0.2226 |  |
|  | cEH | 29 | 0.5517 | 0.5033 | 0.7105 |  |  |  | cEH | 29 | 0.4138 | 0.4235 | 1.0000 |  |
|  | cLP | 31 | 0.3548 | 0.4321 | 0.4026 |  |  |  | cLP | 31 | 0.5484 | 0.5262 | 0.3294 |  |
|  | FOG | 23 | 0.4348 | 0.4638 | 1.0000 |  |  |  | FOG | 23 | 0.6364 | 0.5116 | 0.3907 |  |
|  |  |  |  |  |  |  |  |  |  |  |  |  |  |  |
| **Locus** | **Pop** | ***N*** | **H_O_** | **H_E_** | **P_HWE_** |  |  | **Locus** | **Pop** | ***N*** | **H_O_** | **H_E_** | **P_HWE_** |  |
| Hti65^3^ | IRA | 31 | 0.4839 | 0.5833 | 0.2790 |  |  | Heu72^3^ | IRA | 31 | 0.2333 | 0.4226 | 0.0039 | ** |
| F_ST_=0.123 | BUL | 33 | 0.5152 | 0.5240 | 0.5321 |  |  | F_ST_=0.043 | BUL | 33 | 0.4000 | 0.5492 | 0.0016 | ** |
|  | SGR | 17 | 0.5294 | 0.4421 | 1.0000 |  |  |  | SGR | 17 | 0.5625 | 0.5423 | 0.5664 |  |
|  | *CRE | 16 | 0.6250 | 0.7157 | 0.2857 |  |  |  | *CRE | 16 | 0.6875 | 0.5343 | 0.6042 |  |
|  | HUN | 24 | 0.4583 | 0.5098 | 0.4556 |  |  |  | HUN | 24 | 0.3333 | 0.6690 | 0.0010 | ** |
|  | SLK | 45 | 0.3333 | 0.3438 | 0.0880 |  |  |  | SLK | 45 | 0.3864 | 0.6429 | < 0.00015 | ** |
|  | GER | 33 | 0.4849 | 0.4723 | 0.7521 |  |  |  | GER | 33 | 0.2727 | 0.5217 | 0.0036 | ** |
|  | BEL | 18 | 0.3333 | 0.4095 | 0.2785 |  |  |  | BEL | 18 | 0.3333 | 0.6556 | 0.0066 | ** |
|  | ESP | 26 | 0.6154 | 0.6003 | 0.9023 |  |  |  | ESP | 26 | 0.3077 | 0.6124 | < 0.00015 | ** |
|  | SSP | 20 | 0.1053 | 0.1024 | 1.0000 |  |  |  | SSP | 20 | 0.4737 | 0.6060 | 0.5838 |  |
|  | NIT | 33 | 0.5313 | 0.5566 | 0.2158 |  |  |  | NIT | 33 | 0.3448 | 0.6183 | 0.0020 | ** |
|  | *EIT | 25 | 0.1667 | 0.1959 | 0.2032 |  |  |  | *EIT | 25 | 0.2000 | 0.3205 | 0.0321 | ** |
|  | *CIT | 45 | 0.4222 | 0.4320 | 0.5473 |  |  |  | *CIT | 45 | 0.2857 | 0.5734 | < 0.00015 | ** |
|  | *SIT | 28 | 0.4643 | 0.5429 | 0.1249 |  |  |  | *SIT | 28 | 0.4286 | 0.4805 | 0.0953 |  |
|  | *SIC | 23 | 0.4348 | 0.5478 | 0.1684 |  |  |  | *SIC | 23 | 0.3044 | 0.6319 | < 0.00076 | ** |
|  | *PAN | 41 | 0.7561 | 0.7576 | 0.8665 |  |  |  | *PAN | 41 | 0.1852 | 0.1761 | 1.0000 |  |
|  | *MAL | 29 | 0.6897 | 0.6515 | 0.3859 |  |  |  | *MAL | 29 | 0.1379 | 0.1918 | 0.2415 |  |
|  | *TUN | 22 | 0.4762 | 0.5575 | 0.4289 |  |  |  | *TUN | 22 | 0.3810 | 0.5389 | 0.0338 |  |
|  | *MOR | 20 | 0.5500 | 0.6667 | 0.0084 |  |  |  | *MOR | 20 | 0.3125 | 0.3851 | 0.1331 |  |
|  | cFV | 24 | 0.5000 | 0.5683 | 0.5970 |  |  |  | cFV | 24 | 0.2917 | 0.3590 | 0.1661 |  |
|  | cGC | 80 | 0.6000 | 0.6201 | 0.0118 |  |  |  | cGC | 80 | 0.2692 | 0.2961 | 0.0043 |  |
|  | cTF | 32 | 0.5313 | 0.4995 | 1.0000 |  |  |  | cTF | 32 | 0.0968 | 0.2628 | 0.0011 | ** |
|  | cLG | 19 | 0.6842 | 0.5562 | 0.6139 |  |  |  | cLG | 19 | 0.1667 | 0.2587 | 0.0673 |  |
|  | cEH | 29 | 0.6552 | 0.6576 | 0.8042 |  |  |  | cEH | 29 | 0.1429 | 0.2578 | 0.0408 |  |
|  | cLP | 31 | 0.5807 | 0.6711 | 0.4515 |  |  |  | cLP | 31 | 0.2258 | 0.2618 | 0.0854 |  |
|  | FOG | 23 | 0.4783 | 0.5401 | 0.3000 |  |  |  | FOG | 23 | 0.0435 | 0.3952 | < 0.00015 | ** |
| Hti66^3^ | IRA | 31 | 0.4839 | 0.4744 | 1.0000 |  |  | Heu76^3^ | IRA | 31 | 0.2917 | 0.7199 | < 0.00015 | ** |
| F_ST_=0.075 | BUL | 33 | 0.2424 | 0.3730 | 0.0590 |  |  | F_ST_=0.034 | BUL | 33 | 0.3871 | 0.5933 | 0.0027 | ** |
|  | SGR | 17 | 0.3529 | 0.3832 | 1.0000 |  |  |  | SGR | 17 | 0.4118 | 0.7718 | 0.0087 | ** |
|  | *CRE | 16 | 0.3750 | 0.5585 | 0.0155 |  |  |  | *CRE | 16 | 0.2857 | 0.3730 | 0.2332 |  |
|  | HUN | 24 | 0.2917 | 0.4034 | 0.2917 |  |  |  | HUN | 24 | 0.3750 | 0.5851 | 0.0043 | ** |
|  | SLK | 45 | 0.4222 | 0.4057 | 1.0000 |  |  |  | SLK | 45 | 0.2439 | 0.5089 | < 0.00015 | ** |
|  | GER | 33 | 0.5455 | 0.4434 | 0.6253 |  |  |  | GER | 33 | 0.4000 | 0.8090 | < 0.00015 | ** |
|  | BEL | 18 | 0.3333 | 0.2937 | 1.0000 |  |  |  | BEL | 18 | 0.6667 | 0.6857 | 0.4545 |  |
|  | ESP | 26 | 0.5000 | 0.4193 | 0.2413 |  |  |  | ESP | 26 | 0.3750 | 0.7323 | < 0.00015 | ** |
|  | SSP | 20 | 0.4000 | 0.4718 | 0.0029 |  |  |  | SSP | 20 | 0.3000 | 0.6359 | < 0.00015 | ** |
|  | NIT | 33 | 0.3939 | 0.3865 | 0.4130 |  |  |  | NIT | 33 | 0.3871 | 0.7462 | < 0.00015 | ** |
|  | *EIT | 25 | 0.2000 | 0.3012 | 0.1429 |  |  |  | *EIT | 25 | 0.5600 | 0.7322 | 0.0720 |  |
|  | *CIT | 45 | 0.6000 | 0.4896 | 0.2160 |  |  |  | *CIT | 45 | 0.2500 | 0.7611 | < 0.00015 | ** |
|  | *SIT | 28 | 0.3929 | 0.4682 | 0.6000 |  |  |  | *SIT | 28 | 0.4546 | 0.7643 | 0.0020 | ** |
|  | *SIC | 23 | 0.5217 | 0.4329 | 0.6191 |  |  |  | *SIC | 23 | 0.3000 | 0.6603 | < 0.00076 | ** |
|  | *PAN | 41 | 0.5366 | 0.5724 | 0.4109 |  |  |  | *PAN | 41 | 0.2121 | 0.6429 | < 0.00015 | ** |
|  | *MAL | 29 | 0.5172 | 0.4217 | 0.3716 |  |  |  | *MAL | 29 | 0,0000 | 0.5621 | < 0.00015 | ** |
|  | *TUN | 22 | 0.5000 | 0.4598 | 1.0000 |  |  |  | *TUN | 22 | 0.2105 | 0.5861 | < 0.00076 | ** |
|  | *MOR | 20 | 0.4000 | 0.4667 | 0.6330 |  |  |  | *MOR | 20 | 0,0000 | 0.5704 | < 0.00015 | ** |
|  | cFV | 24 | 0.5217 | 0.5101 | 1.0000 |  |  |  | cFV | 24 | 0,0000 | 0.7158 | < 0.00015 | ** |
|  | cGC | 80 | 0.5875 | 0.5024 | 0.1791 |  |  |  | cGC | 80 | 0.1035 | 0.5572 | < 0.00015 | ** |
|  | cTF | 32 | 0.6250 | 0.4901 | 0.1538 |  |  |  | cTF | 32 | 0.2000 | 0.6579 | 0.0068 | ** |
|  | cLG | 19 | 0.4737 | 0.4225 | 1.0000 |  |  |  | cLG | 19 | 0,0000 | 0.7879 | 0.0011 | ** |
|  | cEH | 29 | 0.6207 | 0.4791 | 0.1327 |  |  |  | cEH | 29 | 0,0000 | 0.5455 | 0.0120 | ** |
|  | cLP | 31 | 0.2759 | 0.4356 | 0.0757 |  |  |  | cLP | 31 | 0.1875 | 0.6754 | < 0.00015 | ** |
|  | FOG | 23 | 0.2609 | 0.3952 | 0.1224 |  |  |  | FOG | 23 | 0.0588 | 0.0588 | 1.0000 |  |

**Table S3.** Pairwise population differentiation (F_ST_) based on microsatellite data.

F_ST_-values coloured according to inset legend; insignificant values (*p* < 0.05) indicated in light grey italic. Population abbreviations correspond to Fig. 1 (* = putative hybrid populations) and their colouration corresponds to the predominant assignment of the population’s samples to the *K*=2 Structure clusters (see Fig. 6a) *H. tithymali* (red) and *H. euphorbiae* (blue). Green box highlights F_ST_ values between the populations of these two clusters.


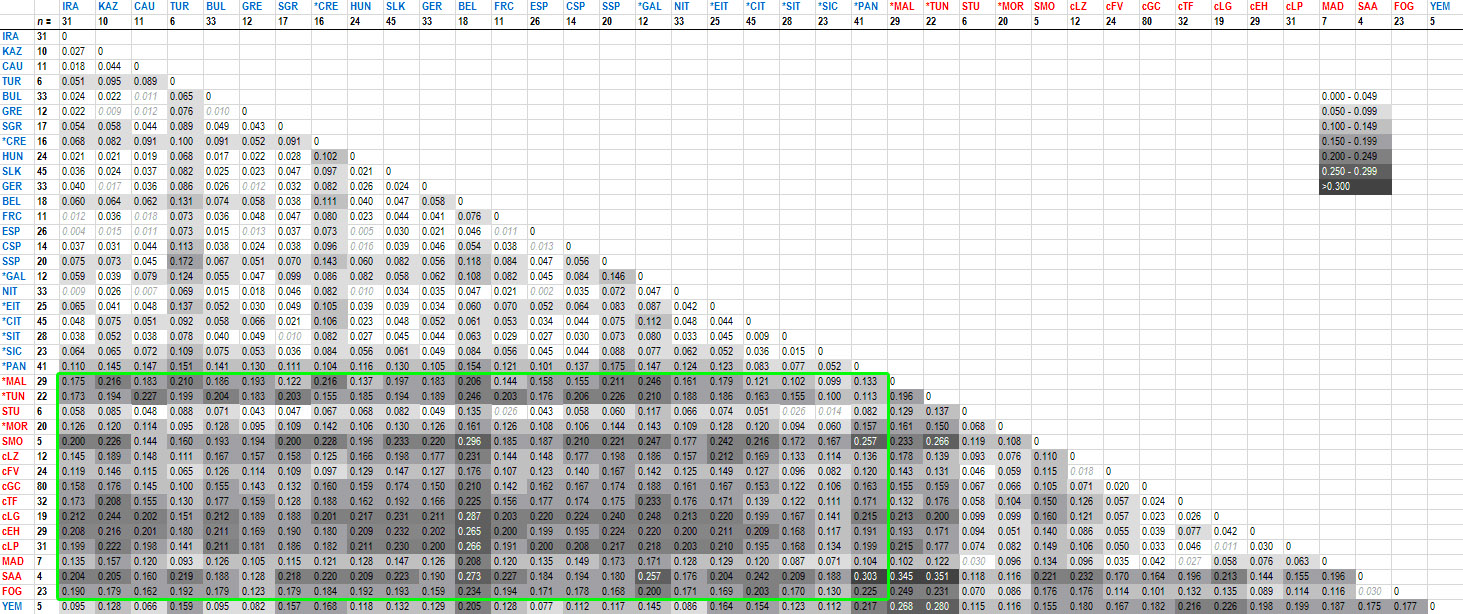


**Supplementary figures**


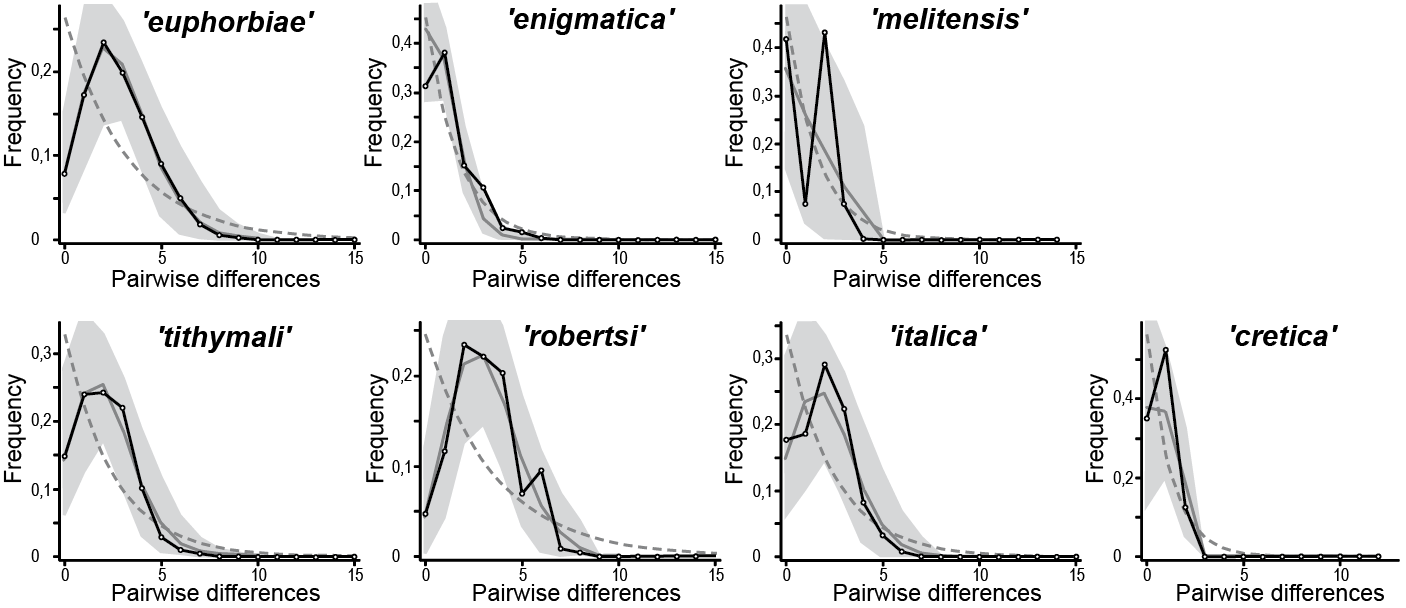


**Figure S1.** Mismatch analyses for mitochondrial haplogroups.

Frequency distributions of pairwise differences of mtDNA sequences are depicted for each haplogroup. The black line depicts the observed frequency distribution, the dashed grey line the expected distribution under the population expansion model, and the solid grey line the model fitted to the data by Arlequin with the light grey area indicating the 95 % confidence interval.


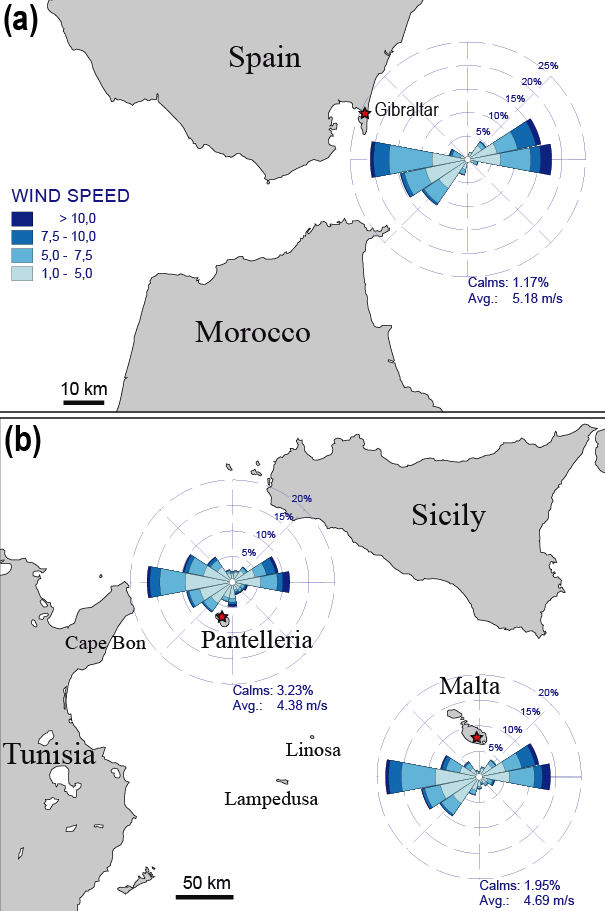


**Figure S2.** Wind roses for the Strait of Sicily (a) and Strait of Gibraltar (b).

Wind roses represent wind direction and speed [see *inset* legend in (a)] measured in the main season and daytime of the HEC’s flight activity (March – October, 10 p.m. – 1 a.m. CET) in 2001-2009 at the stations #08495 (Gibraltar) (a), #16470 (Pantelleria) and #16597 (Malta, Luqa) (b); data from the ‘National Climate Data Center’ of the NOAA (http://www.ncdc.noaa.gov/cdo-web/search; accessed 06/10/2013), compiled with WRPLOT View (Lakes Environmental Software). Maps: ArcGIS version 10, ESRI (http://www.esri.de).

**Supplementary References**

1. Mende, M. B. & Hundsdoerfer, A. K. Mitochondrial lineage sorting in action – historical biogeography of the *Hyles euphorbiae* complex (Sphingidae, Lepidoptera) in Italy. BMC Evol. Biol. 13, 83. (2013).

2. Hundsdoerfer, A. K., Sanetra, M., Corbeil, D. & Stuckas, H. Eleven hawkmoth microsatellite loci of Canary Island *Hyles tithymali* (Lepidoptera). Cons. Gen. Res. 2, 241–244. (2009).

3. Mende, M. B., Stuckas, H. & Hundsdoerfer, A. K. Eight new microsatellite loci of the Western Palearctic *Hyles euphorbiae* complex (Lepidoptera, Sphingidae). Ann. Zool. Fenn. 48, 142–146. (2011).
